# Enabling High‐Boiling‐Point Green Solvent Recycling Using Organic Solvent Nanofiltration Membranes

**DOI:** 10.1002/cssc.202501117

**Published:** 2025-09-23

**Authors:** Marjolaine Thomas, Ana R. Nabais, Maria J. Burggraef, Ludmila Peeva, Jane Murray, Andrew G. Livingston

**Affiliations:** ^1^ School of Engineering and Materials Science Queen Mary University of London London E1 4NS UK; ^2^ Merck KGaA Frankfurter Straße 250 64293 Darmstadt Germany

**Keywords:** green chemistry, green solvents, membranes, organic solvent nanofiltration, solvent recovery

## Abstract

The extensive use of organic solvents in industrial processes contributes to environmental pollution, resource depletion, and human health hazards, incentivizing the development of more sustainable alternatives. In response, bio‐based “green” solvents have emerged as environmentally benign substitutes, offering reduced toxicity and lower carbon footprints. However, their industrial implementation is challenged by high viscosity, energy‐intensive recovery, and limited life‐cycle sustainability when employed in single‐use processes. Herein, organic solvent nanofiltration (OSN) as an energy‐efficient and selective membrane‐based technology for recycling green solvents is explored. OSN membranes enable solvent purification by selectively removing impurities while preserving solvent integrity, reducing waste generation and energy consumption. OSN performance in the recovery of widely used green solvents, including acetone, acetonitrile, ethyl acetate, Cyrene, dimethyl isosorbide (DMI), and γ‐valerolactone (GVL), is evaluated. The potential of OSN for in‐process solvent recycling is demonstrated, with a case study on the recycling of Cyrene in synthetic leather production, demonstrating the feasibility of OSN membranes in maintaining solvent purity and enabling a more sustainable manufacturing process.

## Graphical abstract

This study demonstrates the use of organic solvent nanofiltration (OSN) membranes for the efficient recovery and recycling of bio‐based green solvents. The work highlights OSN's potential as a scalable, energy‐efficient technology to support sustainable industrial solvent management.

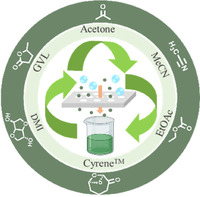

## 1 Introduction

Organic solvents play a critical role in modern industry, prized for their ability to dissolve materials and mediate chemical reactions.^[^ ^1^ ^]^ These properties have made them indispensable in applications such as chemical synthesis and pharmaceutical applications. However, the extensive use of organic solvents is accompanied by significant environmental challenges, including their toxicity and reliance on nonrenewable hydrocarbon origins. Addressing these issues has become a key focus in the pursuit of greener, more sustainable industrial processes.^[^ ^2^ ^]^

Recent advances have introduced “green solvents”—solvents derived from biological sources—as eco‐friendly alternatives to conventional hydrocarbon‐based solvents.^[^ ^3^ ^]^ These bio‐based solvents offer lower toxicity and reduced environmental impact, positioning them as promising substitutes for their conventional counterparts.^[^ ^4^ ^]^ Examples of green solvents include 2‐methyltetrahydrofuran (2‐MeTHF),^[^ ^5^ ^]^ Cyrene,^[^ ^4^, ^6^ ^]^ dimethyl isosorbide (DMI),^[^ ^7^ ^]^ and γ‐valerolactone (GVL).^[^ ^8^ ^]^ Despite these solvents’ growing popularity, which represents a significant step forward in reducing the environmental footprint of industrial processes, challenges remain regarding their life‐cycle sustainability.^[^ ^9^ ^]^

A critical issue is that green solvents are often utilized in single‐use processes, leading to increased waste and environmental burden. While in‐process recycling could improve their sustainability, traditional recovery methods such as distillation are often inefficient, particularly for green solvents with high boiling points. As a result, alternative separation technologies that enable efficient solvent recycling while minimizing energy consumption are urgently needed.

Membrane technology plays an essential role in sustainable industrial practices due to its low environmental footprint during operation compared to conventional thermal separation methods.^[^ ^10^ ^]^ Over the past few decades, membrane‐based separations have gained significant attention for their exceptional performance in water and solvent treatment, gas separation, and other areas, including air purification, biomedical applications, energy production, food processing, and chemical separations.^[^ ^11^ ^]^ Furthermore, membrane technology has also been widely recognized as a “green” separation technology because of its low energy consumption, high selectivity, and role in purification without creating additional waste streams.^[^ ^12^ ^]^

Unlike conventional liquid membrane separation processes which are typically limited to aqueous systems, organic solvent nanofiltration (OSN) operates in organic media, using a pressure gradient to achieve separation of solutes ranging from 50 to 2000 g mol^−1^.^[^ ^13^ ^]^ The retention of organic species in solution is largely governed by the nominal molecular weight cut‐off (MWCO) of the membranes and the molecular size of the solutes, with OSN membranes generally having effective pore sizes in the 1–2 nm range and MWCOs between 100 and 1000.^[^ ^14^ ^]^ Solvent‐resistant membranes with controlled MWCO ensure stable performance across diverse solvents while maintaining a defect‐free morphology.^[^ ^15^ ^]^ OSN offers high efficiency, lower energy consumption, and compatibility with complex solvent mixtures, making it particularly attractive for industries such as petrochemicals, pharmaceuticals, chemicals, and food processing. Additionally, OSN can be integrated into hybrid separation systems alongside distillation and chromatography to enhance solvent purification and recycling evaporation efficiency.^[^ ^15^, ^16^ ^]^

However, to date, the use of membrane technology in connection with green solvents has largely been limited to the use of green solvents in membrane fabrication,^[^ ^17^ ^]^ rather than the use of membranes in processes employing green solvents. In contrast, only a few studies have explored the performance of OSN membranes directly in bio‐based solvents, including 2‐methyltetrahydrofuran (Me‐THF), where membranes were used for the recovery of catalysts.^[^ ^18^ ^]^ These examples illustrate early progress in this area but also underline the clear need for broader investigation of OSN technologies in green solvent systems. Compared with ceramic membranes, polymer membranes offer advantages such as lower production costs, ease of manufacturing, and the ability to tune membrane properties across a wider range of parameters.^[10c]^

**Figure 1:**
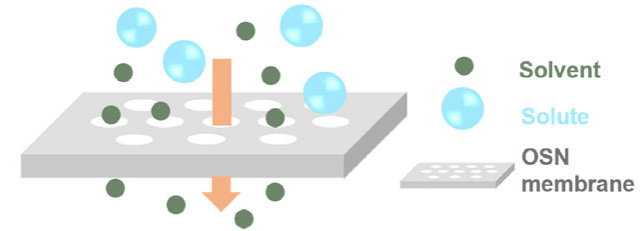
Schematic representation of size‐exclusion‐based OSN membrane separation.

Importantly, the development and application of such membranes resonates with the “12 Principles of Green Membrane Materials and Processes,” which emphasize efficiency, circularity, and reduced environmental impact in alignment with the United Nations’ Sustainable Development Goals.^[^ ^19^ ^]^ These advantages make polymer‐based OSN membranes particularly attractive for addressing the challenges associated with green solvent recycling, while contributing to more sustainable industrial processes (**Figure** **1**).^[15a]^

Here, we investigate the potential of OSN membrane‐based technologies for recycling green solvents, demonstrating their potential to enhance the life‐cycle sustainability of solvent‐intensive industries. By leveraging OSN's selective separation capabilities, we assess the recovery of widely used bio‐based solvents, including acetone, acetonitrile, ethyl acetate, Cyrene, DMI, and GVL.

In particular, we provide an in‐depth case study on synthetic leather production, where OSN enables the efficient recycling of Cyrene, ensuring solvent purity while reducing the energy demand compared to distillation. This example highlights how OSN can directly support greener industrial manufacturing practices.

## 2 Results and Discussion

A range of different green solvents was used in this study to assess their potential for recycling using commercial polymeric OSN membranes. Their characteristics can be found in **Table** **1**. The selected solvents ranged from low boiling point and low viscosity (acetone, acetonitrile, and ethyl acetate) to high boiling point and high viscosity (Cyrene, dimethyl isosorbide, and γ‐valerolactone). Commercial OSN membranes from different suppliers were used to evaluate their capability to recycle these green solvents (**Table** **2** for their characteristics). To evaluate the potential of membrane technology for green solvent recovery, flux and solute rejection measurements were conducted using a cross‐flow filtration setup (see Materials and Methods and Figure S1, Supporting Information).^[^ ^20^ ^]^ The performance of these commercial OSN membranes was investigated in the green solvents, here divided into two sections: low‐boiling‐ and high‐boiling‐point green solvents.

**Table 1:** Characteristics of green solvents used in this work.

Solvents	Structure	MW [g mol–^1^]	Density [g mL^−1^]	Boiling point [°C]	Viscosity [cP] 25 °C	Polarity	Hansen solubility parameters [MPa^1/2^]		
							*δ*_d_	*δ*_p_	*δ*_h_
Acetone	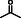	58.08	0.784	56	0.306	0.355	15.50	10.40	7.00
Acetonitrile (MeCN)	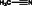	41.053	0.786	8	0.343	0.46	15.30	18.00	6.10
Ethyl acetate (EtOAc)	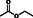	88.106	0.902	77	0.426	0.228	15.80	5.30	7.20
Cyrene	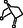	128.13	1.25	226	14.5	0.333	18.7	10.5	6.9
Dimethyl isosorbide (DMI)	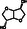	174.19	1.15	94	6.8	0.56	17.6	7.1	7.5
γ‐Valerolactone (GVL)	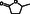	100.116	1.05	207	1.86	0.301	16.7	14	8

**Table 2:** Summary of commercially available polymeric OSN membranes used in this study.

Supplier	Series name	MWCO [Da]a)	Materials and type	Chemical resistancea)	Pressure max [bar]
BORSIG Membrane Technology GmbH	oNF‐1 oNF‐2 oNF‐3	600 350 900	PDMS layer on PAN, TFCb)	Alkanes, aromatics, alcohols, ethers, ketones, esters	35
Evonik MET	PuraMem Flux PuraMem Performance PuraMem Selective	280−600	Cross‐linked PDMS on PAN, TFC	Alcohols, aliphatic hydrocarbons, aromatic hydrocarbons, butyl acetate, ethyl acetate, methyl‐ethyl‐ketone, methyl *tert*‐butyl ether	60
SolSep	NF030105 NF030106 NF030306	300−500 500−1000 500−1000	PDMS	Alcohols, esters, ketones, aromatics, chlorinated, THF	35
AMS (Unisol)	NanoPro S‐3011 NanoPro S‐3012 NanoPro S‐3014	100 180 400	Patented composite film, TFC	Methanol, ethanol, propanol, hexane, THF, acetone, acetonitrile, ethyl acetate, DMF	70
					70
					40

a) According to the manufacturer's information;

b) TFC = thin film composite

### 2.1 Membrane Performance

The average permeance values of membranes measured in the different solvents are presented in **Table** **3**. Higher permeance was generally observed for low‐boiling solvents (acetone and ethyl acetate) due to their lower viscosity and MW, while lower permeance was observed in high‐boiling‐point and high‐viscosity solvents (Cyrene, DMI, and GVL). The performance was the highest for acetone, the solvent with the lowest viscosity (Table 1), ranging from 2.9 to 51 L m^−2 ^h^−1^ bar^−1^ (excluding AMS S‐3014 result), followed by ethyl acetate and acetonitrile with 1.8 to 27 and 0.2 to 12 L m^−2^ h^−1^ bar^−1^, respectively. In contrast, permeance values in GVL, DMI, and Cyrene were lower and ranging from 0.04 to 1.2, 0.04 to 3.9, and 0.04 to 5.6 L m^−2^ h^−1^ bar^−1^, respectively. The AMS series membranes showed no measurable permeance in acetonitrile, Cyrene, DMI, and GVL. Only S‐3014 showed low permeance in acetone and ethyl acetate (0.009 and 0.02 L m^−2^ h^−1^ bar^−1^, respectively). As a result, the AMS series was not considered for the rejection measurements in the high‐boiling‐point green solvents.

**Table 3:** Summary of membrane permeance in different green solvents.

Membranes	Solvent	Permeance [L m^−2^ h^−1^ bar^−1^]	Solvent	Permeance [L m^−2^ h^−1^ bar^−1^]
oNF‐1 oNF‐2 oNF‐3	Acetone	4.6 ± 0.2 2.9 ± 0.1 4.0 ± 0.5	Cyrene	0.04 ± 0.01 0.05 ± 0.01 0.3 ± 0.1
PuraMem Flux PuraMem Performance PuraMem Selective		51.0 ± 1.0 4.7 ± 0.1 5.6 ± 2.0		1.3 ± 0.1 0.06 ± 0.01 0.04 ± 0.01
NF030105 NF030106 NF030306		4.6 ± 0.1 5.8 ± 0.4 3.7 ± 0.5		0.06 ± 0.01 5.6 ± 0.1 1.3 ± 0.1
NanoPro S‐3011 NanoPro S‐3012 NanoPro S‐3014		– – 0.009 ± 0.006		– – –
oNF‐1 oNF‐2 oNF‐3	Acetonitrile (MeCN)	1.5 ± 0.1 0.4 ± 0.2 0.7 ± 0.5	Dimethyl isosorbide (DMI)	0.2 ± 0.1 0.06 ± 0.01 0.5 ± 0.1
PuraMem Flux PuraMem Performance PuraMem Selective		12.0 ± 0.6 1.8 ± 0.1 3.0 ± 1.0		3.9 ± 2.0 0.3 ± 0.1 0.16 ± 0.01
NF030105 NF030106 NF030306		1.0 ± 0.6 3.5 ± 0.4 0.2 ± 0.1		0.09 ± 0.01 0.04 ± 0.01 0.04 ± 0.02
NanoPro S‐3011 NanoPro S‐3012 NanoPro S‐3014		– – –		– – –
oNF‐1 oNF‐2 oNF‐3	Ethyl acetate (EtOAc)	4.6 ± 1.0 3.0 ± 0.1 4.6 ± 0.1	γ‐Valerolactone (GVL)	0.17 ± 0.1 0.06 ± 0.01 0.5 ± 0.1
PuraMem Flux PuraMem Performance PuraMem Selective		27.0 ± 5.0 5.0 ± 2.3 5.6 ± 0.1		1.2 ± 0.1 0.1 ± 0.01 0.05 ± 0.01
NF030105 NF030106 NF030306		5.1 ± 0.1 1.8 ± 0.1 2.9 ± 0.1		0.09 ± 0.01 0.04 ± 0.01 0.04 ± 0.02
NanoPro S‐3011 NanoPro S‐3012 NanoPro S‐3014		– – 0.02 ± 0.01		– – –

The permeance for each membrane and each solvent was calculated from the average over 3 days from three to four membranes using Equation (1). A constant pressure of 30 bar was used for each test.

**Figure 2:**
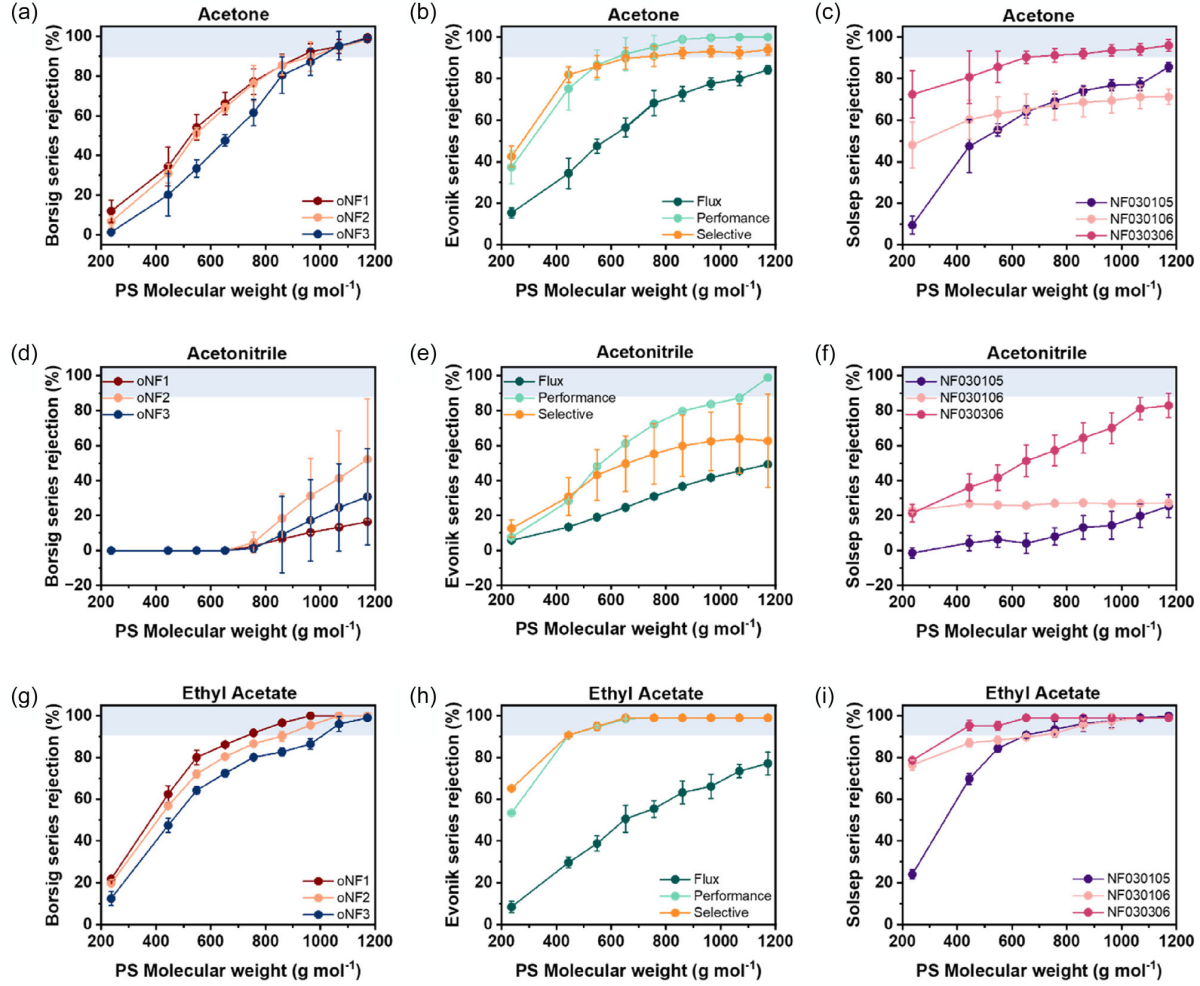
Average membrane rejection over 3 days tested in a–c) acetone, d–f) acetonitrile, and g–i) ethyl acetate. The rejection was measured after 24, 48, and 72 h in different green solvents, at 30 bar, 120 L h^−1^, and 35 °C.

In acetone (**Figure** **2**a–c), high rejection values (*R* > 90%) were achieved by the Borsig series (oNF‐1, oNF‐2, and oNF‐3), PuraMem Selective and Performance (Evonik), and NF030306 (SolSep), with MWCOs of 1070, 950, 1070, 550, 750, and 960 g mol^−1^, respectively. In contrast, PuraMem Flux, NF030105, and NF030106 showed rejections lower than 90% over the whole range of tested MWs. In acetonitrile (Figure 2d–f), low rejection was observed for all membranes, except for PuraMem Performance, which exhibited an MWCO of 950 g  mol^−1^. Ethyl acetate (Figure 2g–i) enabled high rejection for all membranes tested, except PuraMem Flux. Among the AMS membranes, only S‐3014 exhibited measurable rejection in acetone and ethyl acetate; however, no MWCO could be determined after 24 h of testing, indicating limited separation performance (Figure S8, Supporting Information). These data confirm an inverse correlation between permeance and rejection, consistent with the typical flux–selectivity trade‐off observed in membrane separations. MWCO values for each solvent are detailed in Table S1, Supporting Information.

**Figure 3:**
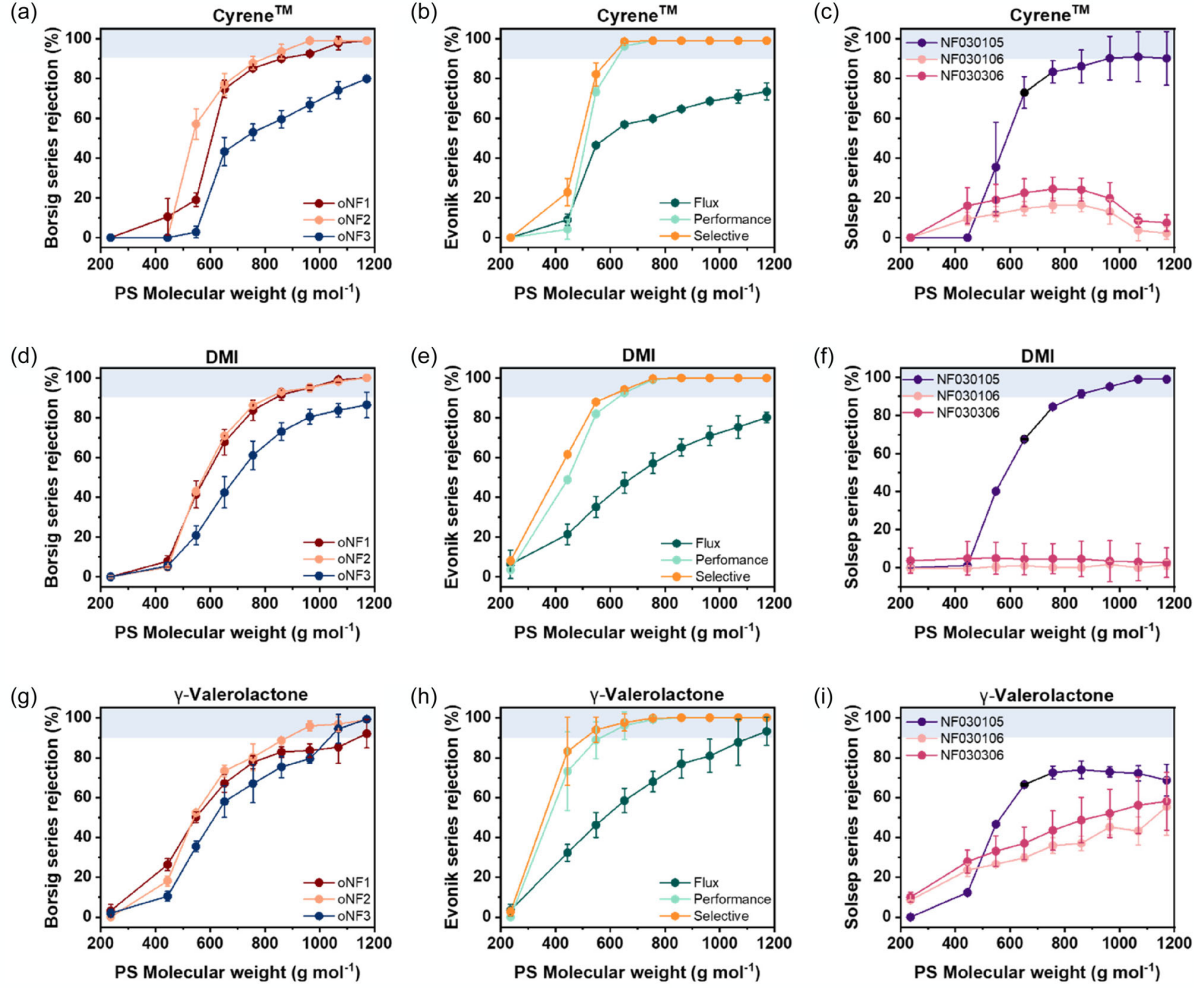
Average membrane rejections over 3 days tested in a–c) Cyrene, d–f) DMI, and g–i) γ‐valerolactone. The rejection was measured after 24, 48, and 72 h in different green solvents at 30 bar, 120 L h^−1^, and 35 °C.

For high‐boiling‐point solvents, similar selectivity trends were observed. In Cyrene (**Figure** **3**a–c), oNF‐1, oNF‐2, PuraMem Performance, and PuraMem Selective achieved > 90% rejection, with MWCOs ranging from 850 to 800 g mol^−1^ (Borsig) and 540 g  mol^−1^ (Evonik). Lower rejection was observed for oNF‐3, PuraMem Flux, and the SolSep series. DMI (Figure 3d–f) produced comparable results, with oNF‐1, oNF‐2, PuraMem Performance and Selective, and NF030105 with a rejection of >90%, with MWCOs consistent with those in Cyrene for Borsig membranes and slightly higher values (600–620 g  mol^−1^) for the Evonik membranes. In GVL (Figure 3g–i), Borsig and Evonik membranes maintained high rejection, except for PuraMem Flux and the SolSep series.

Overall, oNF‐1 and oNF‐2 and PuraMem Performance and Selective membranes demonstrated a good separation performance in all solvents except acetonitrile. The two best performing membranes (PuraMem Performance and Selective) demonstrated the highest rejections in high‐boiling‐point green solvents with the lowest MWCOs. All obtained permeance and polystyrene rejection data measured at 24, 48, and 72 h are shown in Figure S2–S8, Supporting Information.

### 2.2 Membrane Characterization

After the 3 day cross‐flow testing, all membranes were observed and then characterized by scanning electron microscopy (SEM) to assess morphological changes. In terms of visual appearance, in acetone, surface creases were observed for oNF‐2 and oNF‐3, while SEM cross sections confirmed noticeable structural changes for oNF‐1, oNF‐2, and the SolSep series (Figure S9–S12, Supporting Information). In contrast, all membrane series tested in ethyl acetate retained their visual aspects, and SEM images showed minimal deformation, indicating good morphological stability in this solvent (Figure S13–S14, Supporting Information). In acetonitrile, clear defects were identified in several membranes. Surface creases were observed on oNF‐1, PuraMem Performance and Selective, NF030105, and S‐3011, which matched the significant structural changes observed in SEM cross sections. The finger‐like porous structure appeared collapsed after exposure to acetonitrile (Figure S9–S12, Supporting Information).

For the high‐boiling‐point solvents, membranes showed relatively higher stability. In Cyrene and DMI, no visual changes were observed for the Borsig, PuraMem, NF030105 (SolSep), and AMS series. Only NF030106 and NF030306 displayed visible signs of damage, including a black coloration and some delamination (Figure S11, Supporting Information), likely due to interactions between the support and the solvents. SEM cross sections supported these findings, showing only small compaction for oNF‐1 and oNF‐2 after the 72 h test in Cyrene and DMI. In GVL, the PuraMem and AMS series maintained their top‐layer thickness, with no apparent morphological damage. Only minor defects were noted for S‐3012. However, more pronounced structural deformation was observed in the Borsig and SolSep membranes, both visually and in SEM images, confirming reduced membrane stability in this solvent. Detailed characterizations of the membrane behavior in all green solvents can be found in Figure S12–S16 and Table S3, Supporting Information.

### 2.3 Case Study: Recycling of Cyrene for Synthetic Leather Production

Synthetic leather is widely produced using polyurethane (PU) formulations in dimethylformamide (DMF) as the primary solvent. PU is typically synthesized from a mixture of long‐chain polyols (average MW 200–10 000 g mol^−1^) and polyisocyanates (MW 60–4000 g mol^−1^) dissolved in DMF.^[^ ^21^ ^]^ While DMF has been an industry standard due to its ability to effectively solubilize PU components, low cost, and widespread availability, it poses significant health and environmental risks, leading to increasing regulatory restrictions as the European Chemicals Agency (ECHA) has classified DMF under REACH as a substance of very high concern. In addition to its toxicity, large‐scale solvent discharges remain a major issue in PU‐based synthetic leather manufacturing, with global estimates indicating more than one million tons of solvents, including DMF, are discharged annually—representing not only an environmental threat but also economic losses approaching USD 1.8 billion per year. These challenges have prompted increasing interest in safer, greener solvent alternatives and more sustainable process technologies.^[^ ^22^ ^]^ Therefore, efforts toward safer and greener solvent alternatives are being made.^[^ ^23^ ^]^

**Figure 4:**
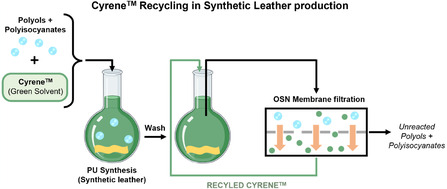
Schematic process of the recycling of Cyrene used in the synthetic leather production using OSN technology.

Recent studies have identified bio‐based green solvents such as Cyrene^[^ ^24^ ^]^ and γ‐valerolactone^[^ ^25^ ^]^ as potential replacements for DMF in PU synthesis. These solvents offer lower toxicity and improved sustainability, aligning with the principles of green chemistry. However, their high viscosity and boiling points present challenges for their efficient recovery and recycling, making conventional distillation‐based solvent recovery energy‐intensive and costly. In a typical synthetic leather production process, after PU synthesis, the reaction mixture contains unreacted polyols and oligomers dissolved in the solvent. Efficient separation of these high‐value polyols from the solvent is crucial to enable solvent reuse and minimize waste. OSN membranes can selectively retain the polyols while allowing the purified solvent to permeate, directly addressing this critical step in the manufacturing process. To address this, membrane‐based OSN presents an energy‐efficient alternative for solvent purification and reuse in synthetic leather manufacturing. Based on the performance data presented in the previous section, four high‐performing OSN membranes were selected for this case study, oNF‐1 and oNF‐2 from the Borsig series and PuraMem Performance and Selective from Evonik (Table S2 and Figure S15, Supporting Information, for further characterizations). They showed promising performances when tested with PS markers (Figure 3). The objective was to separate polyols (main components from synthetic leather production) from Cyrene, enabling solvent recovery while retaining polyol components for reuse. For this study, bio‐based polyols of different MWs (MW = 400–600, 800–1100, and 1800–2200 g mol^−1^) were purchased from Merck and dissolved in a Cyrene solution at 5 g L^−1^ (**Figure** **4**).^[^ ^26^ ^]^

Each membrane was tested in a similar continuous cross‐flow filtration rig at 30 bar operating pressure for 72 h, and two membranes of each type were evaluated. To determine the polyol rejection performance in the membranes, samples were collected from both the feed solution (5 g L^−1^) and the permeate at *t* = 24, 48, and 72 h. Permeate samples were then concentrated at 100 °C under vacuum before high‐performance liquid chromatography (HPLC) analysis. The detailed analytical procedure can be found in the Supporting Information. Given the polydisperse nature of the polyols and peak overlap, peaks could not be assigned in the HPLC spectrum.

**Figure 5:**
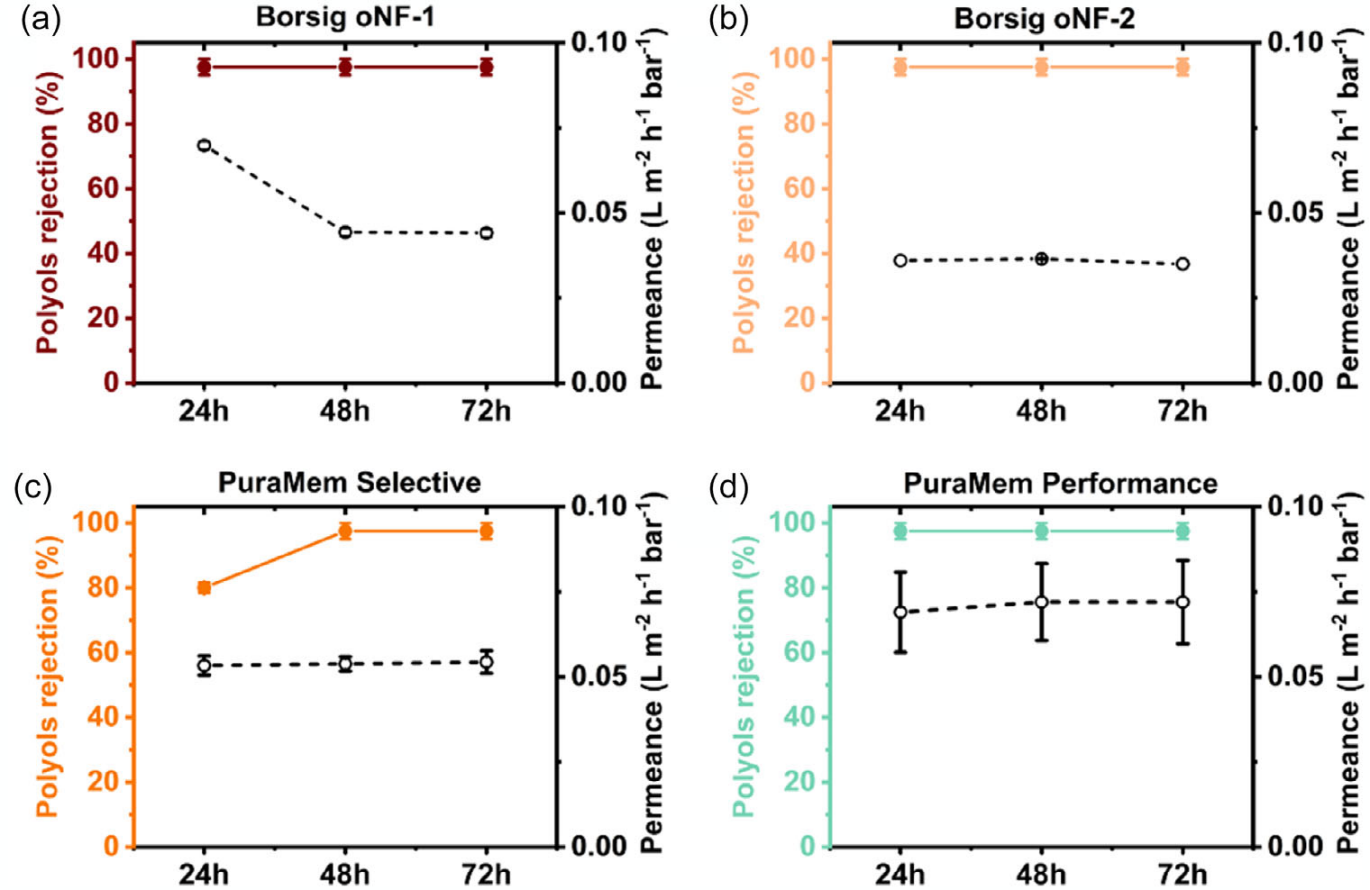
Polyol rejection at 24, 48, and 72 h of a) oNF‐1, b) oNF‐2, c) PuraMem Selective, and d) PuraMem Performance measured in Cyrene at 30 bar, 120 L h^−1^, and 35 °C.

**Figure 6:**
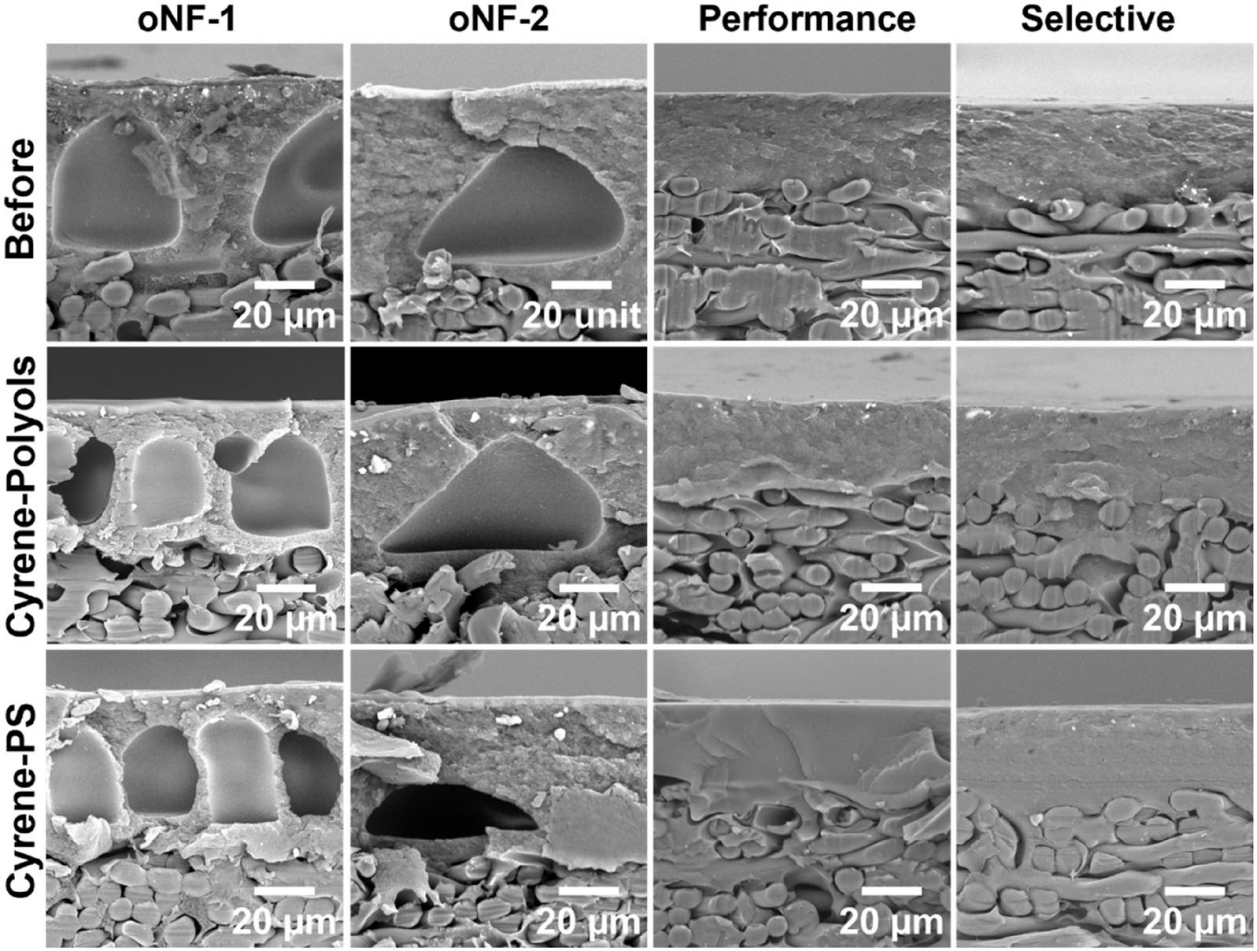
SEM cross‐sectional images of the Borsig oNF‐1, Borsig oNF‐2, PuraMem Performance, and PuraMem Selective membranes after being tested for 3 days.

Following filtration, no detectable peaks were observed on the chromatogram (Figure S19–S23, Supporting Information), suggesting an absence of polyols in the permeate, except for the PuraMem Selective membranes at 24 h (Figure S19, Supporting Information). A rejection of ≥95% after 72 h was achieved for oNF‐1, oNF‐2, and PuraMem Performance membranes and a rejection rate of ≥80% at 24 h, followed by ≥95% at 48 and 72 h for PuraMem Selective (**Figures** **5**a–d). Permeance values for membranes tested in Cyrene with polyols and PS mixtures were comparable within experimental error for oNF‐1, oNF‐2, and PuraMem Performance. PuraMem Selective exhibited slightly higher permeance in Cyrene with polyols compared to the PS mixture one (Figure 5, Table S4, Supporting Information). The membranes were then characterized by SEM, and their support thickness was measured and compared to the membranes before use (**Figure** **6**).

**Figure 7:**
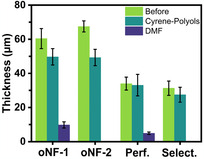
Measured membrane support thickness from SEM cross section before, after 3 day filtration in Cyrene, and immersed in DMF.

The results show a slight membrane compaction after the 3 day test at 30 bar, with similar thicknesses compared to Cyrene–PS, showing good membrane stability in Cyrene overtime. Some fresh membranes were also immersed in DMF for 5 min for stability comparison. The membrane's surfaces were damaged after being soaked in DMF compared to after being tested in Cyrene for 72 h (Figure S17, Supporting Information), especially PuraMem Performance and Selective, where one can see the support layer being dissolved. SEM cross section further confirmed the damage, showing partial to complete dissolution of the polyacrylonitrile (PAN) support beneath the top layers (**Figure** **7**), indicating that DMF is unsuitable as a solvent for OSN applications using these membranes. These results indicate that all four membranes demonstrated effective Cyrene recovery and polyol retention. Notably, oNF‐1, ONF‐2, and PuraMem Performance exhibited higher polyol rejection, while PuraMem Selective showed a lower initial rejection which improved over time.

## 3 Conclusion

The transition to greener industrial practices necessitates not only the adoption of environmentally benign solvents but also the development of sustainable strategies for their reuse and recovery. While bio‐based green solvents such as Cyrene, DMI, and GVL offer a promising alternative to traditional hydrocarbon‐based solvents, their widespread application remains limited by challenges in efficient recycling—especially given their high boiling points and viscosity. This work demonstrates the potential of OSN as a low‐energy, high‐efficiency separation technology capable of overcoming these limitations. By employing polymer‐based OSN membranes, we show that green solvents can be effectively purified and reused without compromising performance or sustainability. The case study on Cyrene recovery within synthetic leather production further highlights OSN's practical applicability and potential for integration into real‐world manufacturing processes. Overall, OSN‐based solvent recovery represents a critical step forward in supporting the circular use of green solvents, reducing industrial carbon footprints, and promoting a more sustainable chemical economy. Future work may explore tailoring membranes for enhanced green solvent resistance, further expanding OSN's potential in sustainable applications.

## 4 Experimental Section

4.1

4.1.1

##### Materials

Acetone, ethyl acetate (EtOAc), acetonitrile (MeCN), and dimethylformamide (DMF) were purchased from Fisher. Cyrene, dimethyl isosorbide (DMI), and γ‐valerolactone (GVL) for solvent screening were purchased from Sigma–Aldrich (UK). The physicochemical properties of the solvents are summarized in Table 1. Polystyrene (PS) dimer (MW = 236 g mol^−1^) was purchased from Sigma–Aldrich, and PS standards 530 and 960 g mol^−1^ from Agilent were used as standards for membrane testing. Bio‐based polyols (400–600, 900–1100, and 1800–2200 g mol^−1^) for membrane testing were purchased from Sigma–Aldrich (UK). Commercial OSN membranes were purchased from different suppliers: Borsig ONF series was purchased from Borsig Membrane Technology GmbH, PuraMem series from Evonik MET, NF series from SolSep, and NanoPro series from AMS (Unisol). Table 2 summarizes the properties of the OSN membranes used. All materials were used as received without further purification.

##### Characterization Methods

SEM images were obtained on Phenom desktop scanning electron microscope from ThermoFisher. Samples for cross section observations were prepared by breaking a piece of membrane and freezing it in liquid nitrogen and placing it between two layers of conductive carbon tape. Approximately 3–5 nm of gold was sputtered onto the top surface of all the samples using a Quorum Q300RT sputter coater. An FEI Inspect F was used for analyzing the samples with an accelerating voltage of less than 5 kV.

##### Membrane Performance

The permeance and rejection of the membranes were determined using cross‐flow filtration. An eight‐cell cross‐flow rig was used, with two parallel paths of four membrane cells each, as described in Scheme S1, Supporting Information. Each membrane had an active area of 14.5 cm^2^, and a minimum of three membranes of each type were tested. All membranes were used as received from the supplier, except for the AMS series, which were presoaked in solvent for at least 6 h prior to use.

The rig operated continuously for at least 72 h at 30 bar at 35 °C, with a cross‐flow rate of 120 L h^−1^ per path. Permeance (*P*) was calculated according to Equation (1):

(1) $$P=\frac{V}{A\cdot t\cdot \Delta p}$$

where *V* is the permeate volume of solvent (L), *A* is the effective membrane area (14.5 × 10^−4^ m^−2^), *t* is time (h), and Δ*p* is the pressure applied during filtration (30 bar). Average permeance was calculated with data from the average of samples taken at 24, 48, and 72 h. The densities and viscosities of all solvents used in permeance tests are detailed in Table 1.

Rejection values were determined for both polystyrene (PS) and polyol samples to assess the membrane separation efficiency. Separation efficiency (*R*) was calculated using Equation (2):

(2) $$R\left(\%\right)=\left[\text{1‐}\frac{C_{p}}{C_{0}}\right]\cdot \text{100 }$$

where *C*_0_ and *C*_p_ are the solute concentrations in the feed and permeate, respectively, in g  L^−1^. For PS testing, concentrations of 0.1 g  L^−1^ for the dimer and 1 g  L^−1^ for PS 530 and PS 960 were used.^[^ ^27^ ^]^ For polyols, bio‐based polyols with MWs ranging from 400 to 2200 g  mol^−1^ were tested at a concentration of 5 g  L^−1^ in Cyrene.

All samples were analyzed by HPLC using an Agilent 1200 system equipped with a Varian‐385 evaporative light‐scattering detector. An ACE 5 C18‐300 column (250 × 4.6 mm) was used for all analyses. For polystyrene samples, HPLC‐grade tetrahydrofuran (THF) and water served as mobile phases B and A, respectively. For polyol samples, the organic phase was a mixture of acetonitrile and methanol (4:1 v/v), while the aqueous phase consisted of water containing 5 mM ammonium acetate. See Figure S1 and Table S1, Supporting Information, for additional details on HPLC calibration, detection limits, and method validation.

## Supporting Information

The authors confirm that the data supporting this article have been included as port of the SI. And raw data will be made available on request.

## Conflict of Interest

The authors declare no conflict of interest.

## Supporting information

Supplementary Material

## Data Availability

The data that support the findings of this study are available in the supplementary material of this article.
