# Supplementary material for: Enabling High‐Boiling‐Point Green Solvent Recycling Using Organic Solvent Nanofiltration Membranes
Source: ChemSusChem. 2025 Sep 23;18(21):e202501117. doi: 10.1002/cssc.202501117 (PMC12584967; doi:10.1002/cssc.202501117)
Supplement: Supplementary file 1 — Supplementary Material [file CSSC-18-e202501117-s001.pdf]

Supplementary Information

Enabling high boiling point green solvent recycling using OSN membranes

Marjolaine Thomas[a], Ana Rita Nabais[a], Maria J. Burggraef[a], Ludmila Peeva[a], Jane Murray[b] and Andrew G. Livingston*[a]

[a]School of Engineering and Materials Science, Queen Mary University of London, UK.
[b]Merck KGaA, Frankfurter Straße 250, 64293 Darmstadt, Germany.

Materials and methods

Materials

Acetone, ethyl acetate (EtOAc), acetonitrile (MeCN) and dimethyl formamide (DMF) were purchased from VWR (UK). Cyrene™, dimethyl isosorbide (DMI) and γ-valerolactone (GVL) for solvent screening were purchased from Sigma-Aldrich (UK). The physicochemical properties of the solvents are summarised in Table 1. Polystyrene (PS) dimer (Mw = 236 g/mol) was purchased from Sigma-Aldrich and PS standards 530 and 960 g mol-^{1} from Agilent and were used as standards for membrane testing. Bio-based polyols (400-600, 900-1100, 1800-2200 gmol-1) for membrane testing were purchase from Sigma-Aldrich (UK) (CAS number 345260-48-2). Commercial OSN membranes were purchased from different suppliers: Borsig ONF series was purchased from Borsig Membrane Technology GmbH, PuraMem series from Evonik MET, NF series from Solsep and NanoPro series from AMS (Unisol). Table 2 summarised the properties of the OSN membranes used. All materials were used as received without further purification.

Methods

<u>Performance testing</u>

The flux and rejection of the membranes were determined using cross-flow filtration. An eight-cell ross-flow rig was used, with two parallel paths of four membranes cells each, as described in Scheme S1. Each membrane had an active area of 14.5 cm^2 and a minimum of three membranes of each type were tested. The rejection was measured using 0.1 g L^{-1} for the PS dimer and 1 g L^{-1} for PS 530 and 960. The rig was operated at a cross-flow rate of 120 L h^{-1} per parallel path, 35°C and 30bar for all rejection measurements. Membranes were used as received, except for the AMS series where membranes were soaked in solvents for at least 6 hours before used.

<u>Polystyrene samples analysis</u>

1 ml samples were taken from the feed and the permeate at t = 24 h, 48 h, 72 h. Rejection samples were analysed using an Agilent 1200 stack coupled with an evaporative light scattering detector (ELSD Varian-385). A reversed phase Hichrom Ace column (C18-300, 250mm 4.6mm I.D.) was used. Two mobile phases were utilized. Mobile phase A was an aqueous solution and mobile phase B was HPLC grade THF. The gradient was 25 min isocratic 27% A. A flow rate of 1 mL min^{-1} and a column temperature of 30 °C were used. The ELSD evaporator was set at 40 °C and the nebulizer at 55 °C. The coefficient of variation of this

analysis at 1 g L^{-1} of each solute was less than 1.2%. The osmosis pressure was assumed to be negligible due to the low concentration of solute.

Polyol samples analysis

1 ml samples were taken from the feed and the permeate at t = 24 h, 48 h, 72 h. HPLC analysis was performed on the same HPLC instrument as for PS. The permeate samples were concentrated at 100 ℃ under vacuum before analysis and a concentration factor was calculated from the sample weight before and after evaporation (Figure S1). An ACE 5 C18-300 column (250 x 4.6 mm) was used, with HPLC-grade Acetonitrile:Methanol (4:1) as the organic mobile phase (B) and water with 5 mM NH_4OAc as the aqueous (A). The gradient used for the samples was a 40 - 95% B gradient over 30 minutes at a column temperature of 30 ℃, and an injection volume of 10 µL. The detection limit for the used polyols was determined to be 1 g/L.

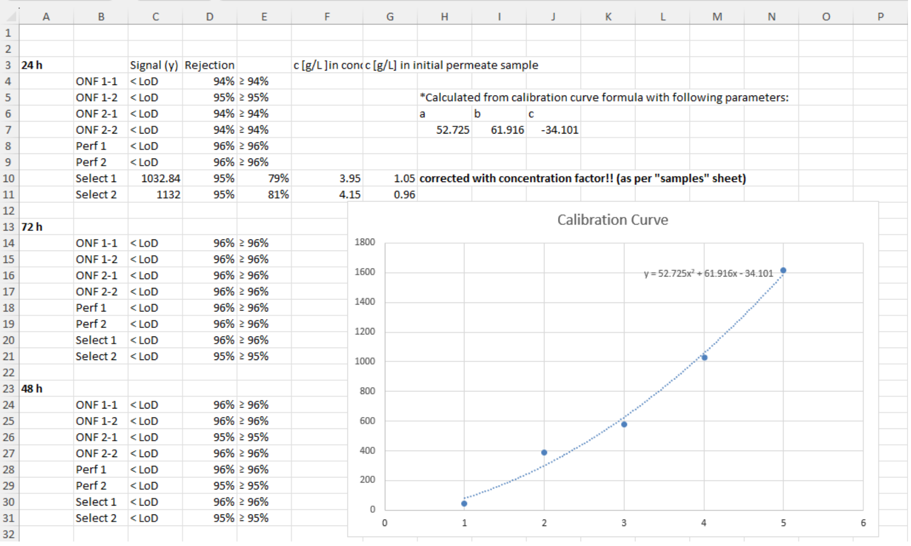

	A	B	C	D	E	F	G	H	I	J
1										
2										
3	**24 h**		Signal (y)	Rejection		c [g/L]in conc	c [g/L] in initial permeate sample			
4		ONF 1-1	< LoD	94%	≥ 94%					
5		ONF 1-2	< LoD	95%	≥ 95%			*Calculated from calibration curve formula with following parameters:		
6		ONF 2-1	< LoD	94%	≥ 94%			a	b	c
7		ONF 2-2	< LoD	94%	≥ 94%			52.725	61.916	-34.101
8		Perf 1	< LoD	96%	≥ 96%					
9		Perf 2	< LoD	96%	≥ 96%					
10		Select 1	1032.84	95%	79%	3.95	1.05	**corrected with concentration factor!! (as per "samples" sheet)**		
11		Select 2	1132	95%	81%	4.15	0.96			
12										
13	**72 h**									
14		ONF 1-1	< LoD	96%	≥ 96%					
15		ONF 1-2	< LoD	96%	≥ 96%					
16		ONF 2-1	< LoD	96%	≥ 96%					
17		ONF 2-2	< LoD	96%	≥ 96%					
18		Perf 1	< LoD	96%	≥ 96%					
19		Perf 2	< LoD	96%	≥ 96%					
20		Select 1	< LoD	96%	≥ 96%					
21		Select 2	< LoD	95%	≥ 95%					
22										
23	**48 h**									
24		ONF 1-1	< LoD	96%	≥ 96%					
25		ONF 1-2	< LoD	96%	≥ 96%					
26		ONF 2-1	< LoD	95%	≥ 95%					
27		ONF 2-2	< LoD	96%	≥ 96%					
28		Perf 1	< LoD	96%	≥ 96%					
29		Perf 2	< LoD	95%	≥ 95%					
30		Select 1	< LoD	96%	≥ 96%					
31		Select 2	< LoD	95%	≥ 95%					
32										

Figure S1. Screenshot of Excel table showing how the rejections for the different membranes were calculated based on a concentration factor.

SEM Characterisation

For scanning electron microscopy (SEM), samples were dried, freeze fractured in liquid nitrogen, and approximately 3–5 nm of gold was sputtered onto the top surface. An FEI Inspect F was used for analysing the samples with an accelerating voltage of 10 kV.

Cross-flow setup

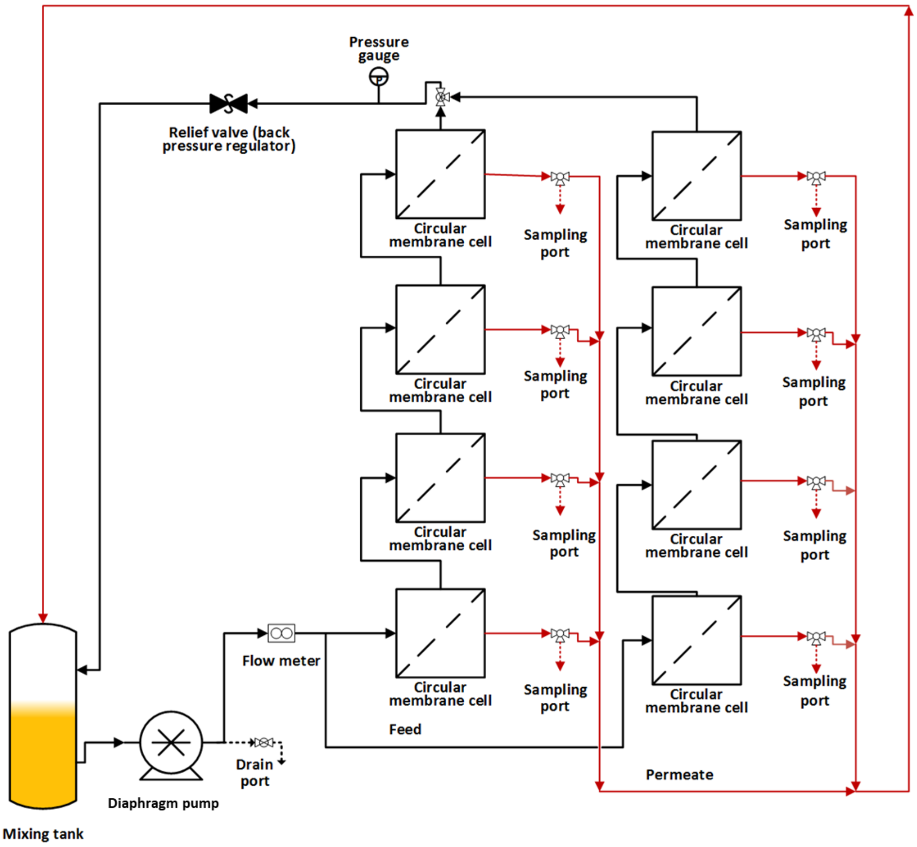

Scheme S1. Schematic representation of cross-flow filtration setup using 8 membranes cell.

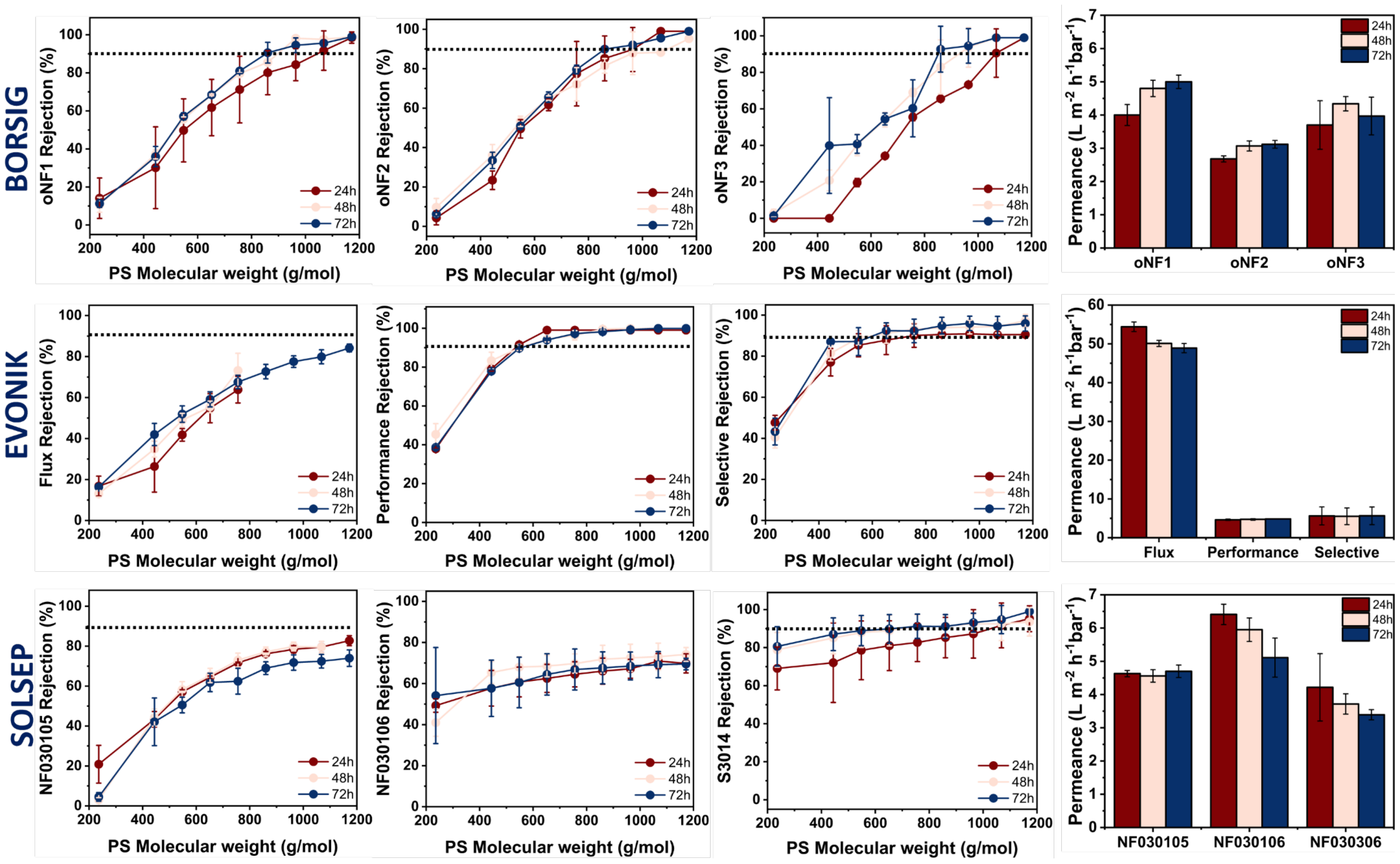

Figure S2. Rejections of different membranes (Borsig, Evonik and Solsep series) tested in **acetone** with corresponding permeance over time at 30bar, 120Lh^{-1}, 35°C.

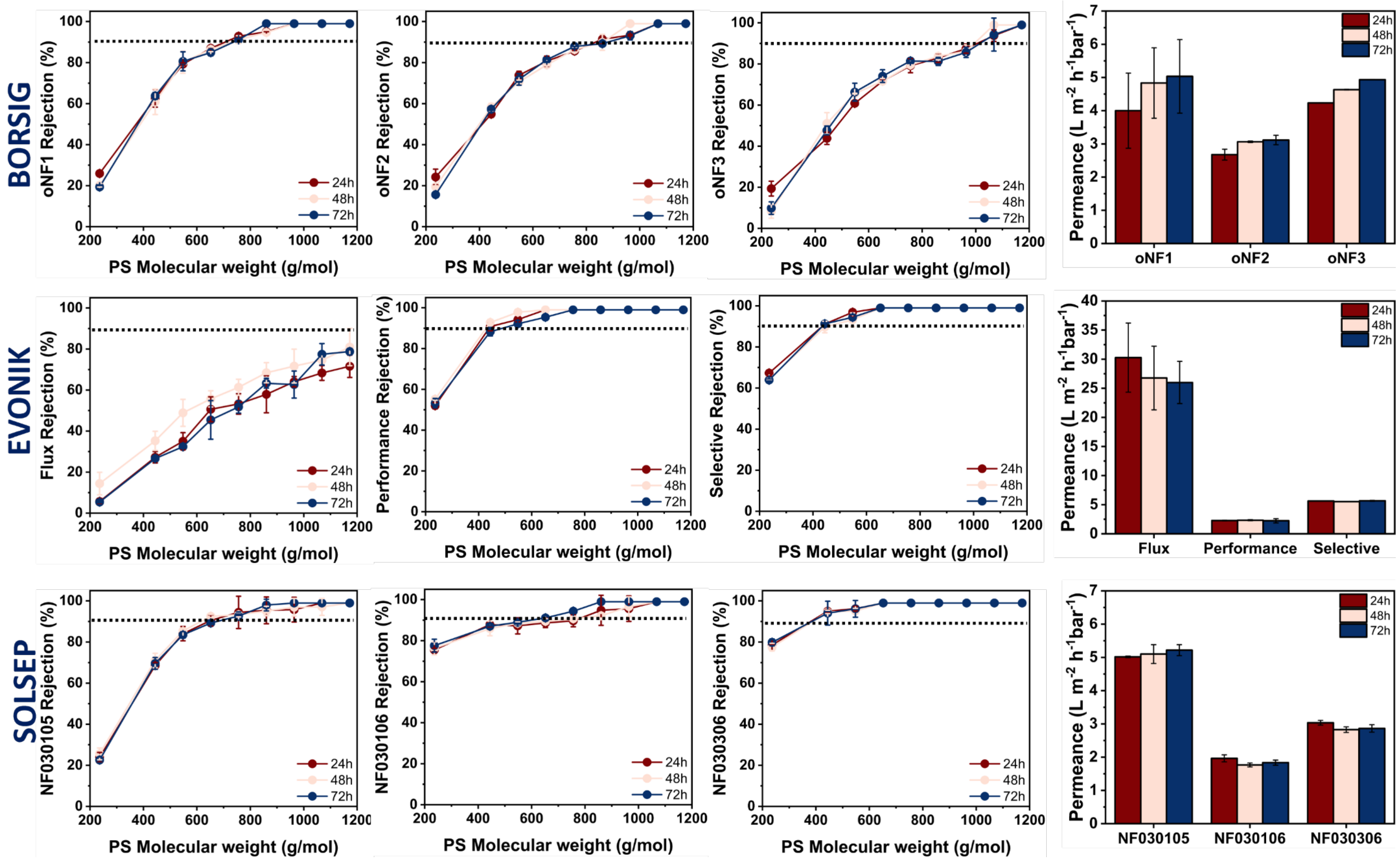

Figure S3. Rejections of different membranes (Borsig, Evonik and Solsep series) tested in **ethyl acetate** with corresponding permeance over time at 30bar, 120Lh^{-1}, 35°C.

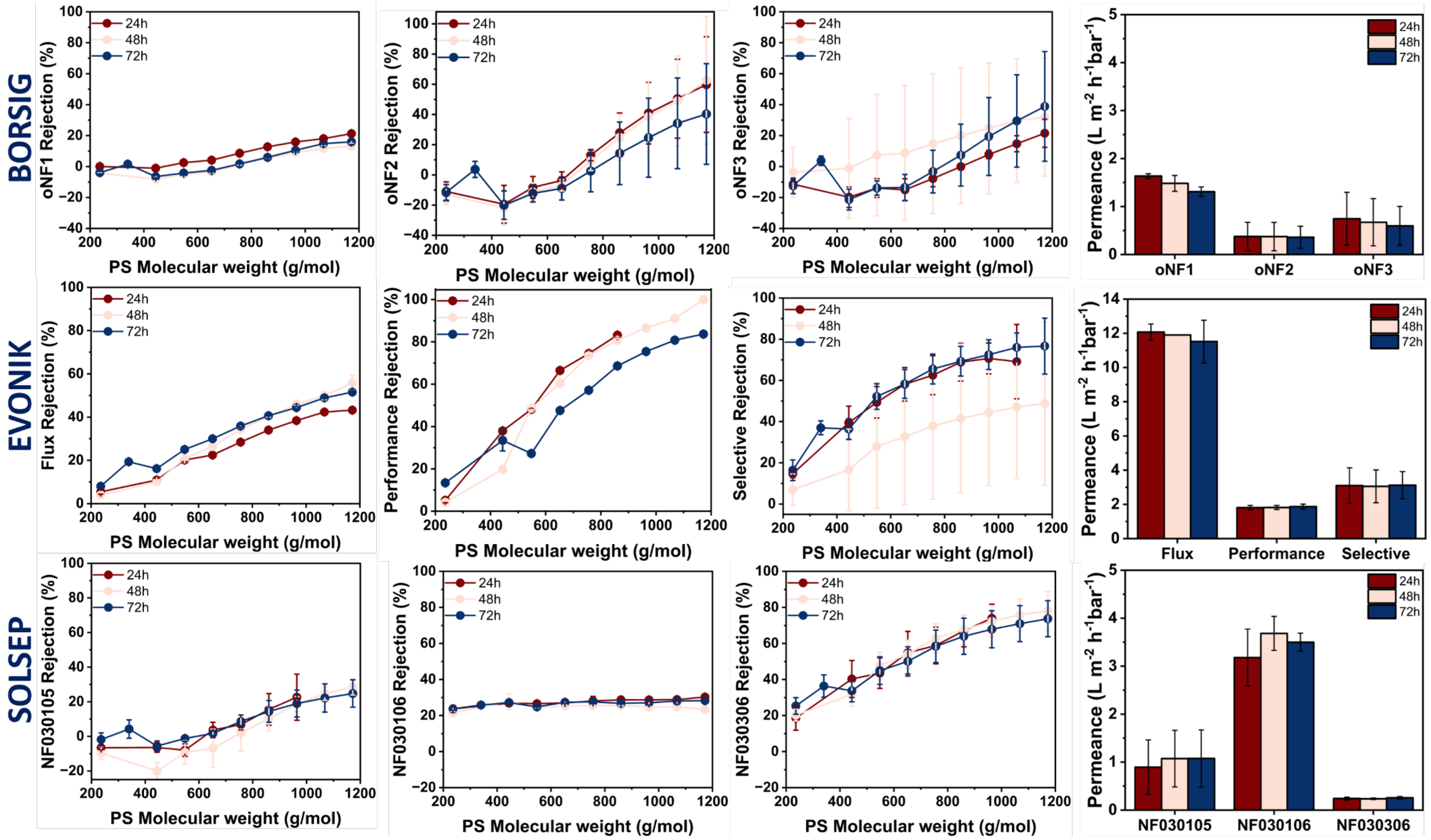

Figure S4. Rejections of different membranes (Borsig, Evonik, Solsep series) tested in **acetonitrile** with corresponding permeance over time at 30bar, 120Lh^{-1}, 35°C.

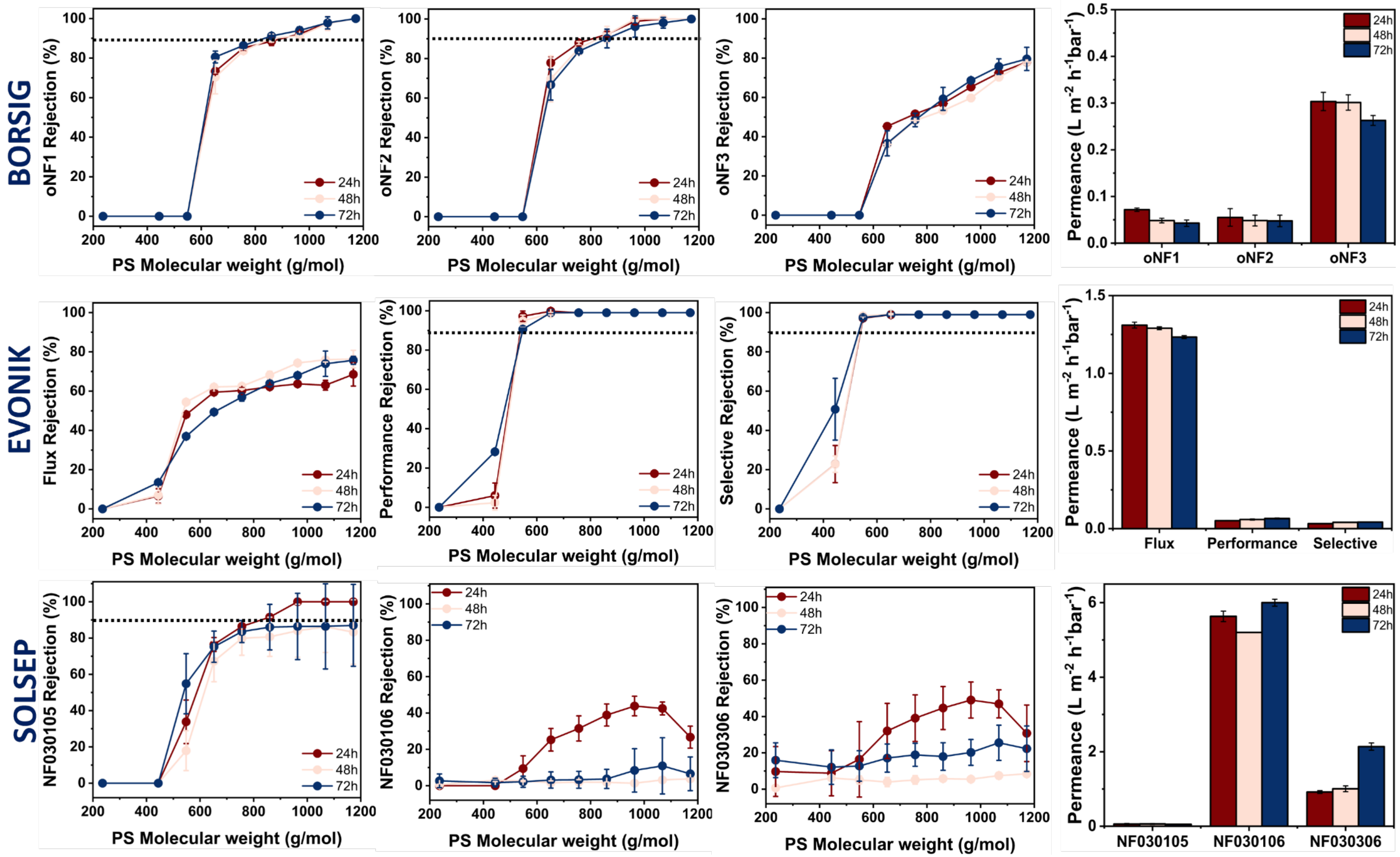

Figure S5. Rejections of different membranes (Borsig, Evonik and Solsep series) tested in **cyrene** with corresponding permeance over time at 30bar, 120Lh^{-1}, 35°C.

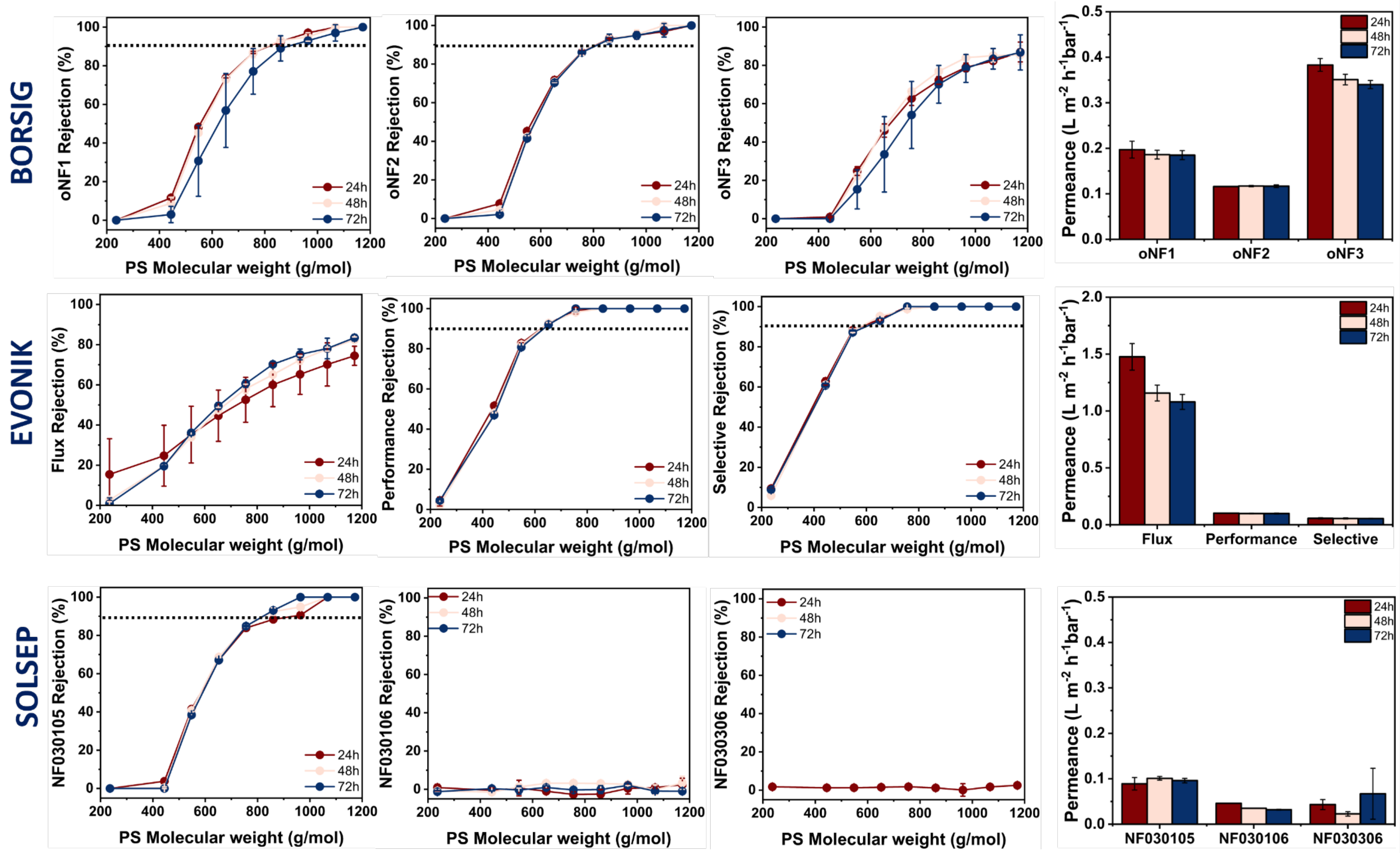

Figure S6. Rejections of different membranes (Borsig, Evonik and Solsep series) tested in **DMI** with corresponding permeance over time at 30bar, 120Lh^{-1}, 35°C.

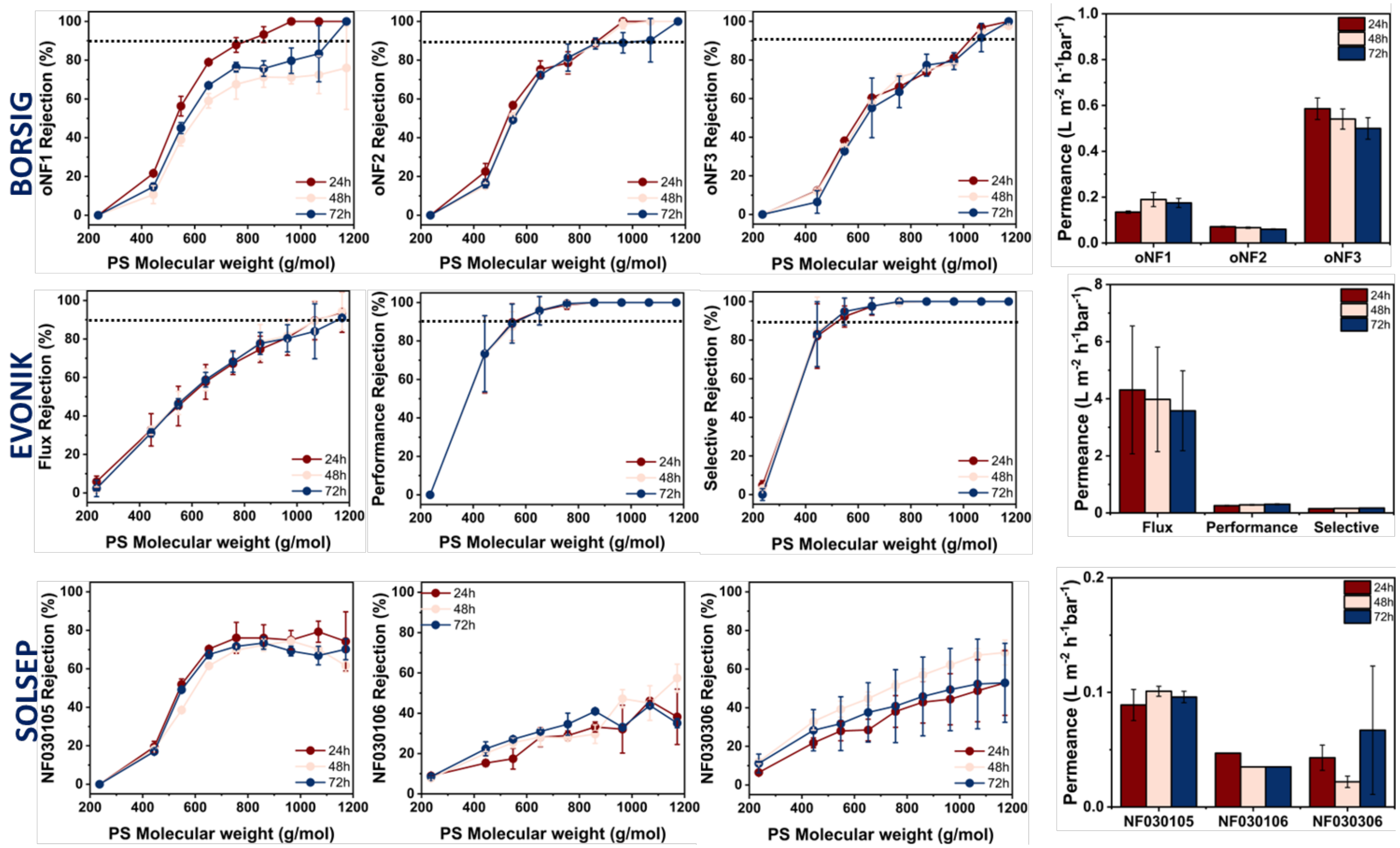

Figure S7. Rejections of different membranes (Borsig, Evonik and Solsep series) tested in **GVL** with corresponding permeance over time at 30bar, 120Lh^{-1}, 35°C.

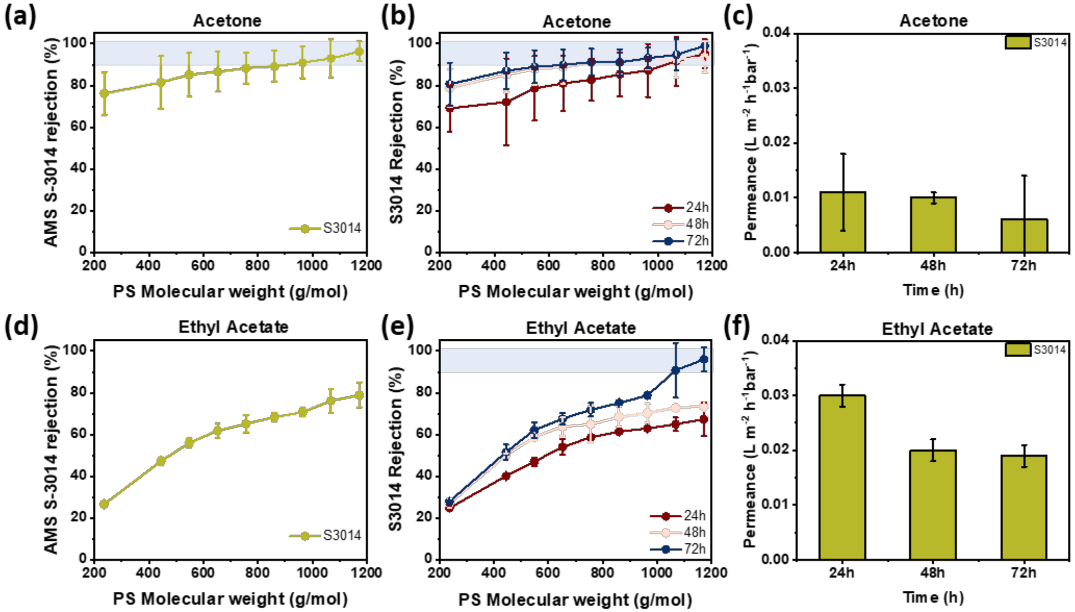

Figure S8. Rejections of different membranes (AMS S-3014) tested in **GVL** with corresponding permeance over time at 30bar, 120Lh^{-1}, 35°C.

Table S1. MWCO at **24h** for the commercial membranes tested in the green solvents for 3 days at 30bar.

Solvents	Membranes	MWCO (g mol^{-1}) at 24h
Acetone	oNF-1	1070
	oNF-2	950
	oNF-3	1070
	PuraMem Flux	-*
	PuraMem Performance	550
	PuraMem Selective	750
	NF030105	1150
	NF030106	-
	NF030306	960
	NanoPro S-3011	-
	NanoPro S-3012	-
	NanoPro S-3014	1000
Ethyl Acetate	oNF-1	780
	oNF-2	850
	oNF-3	1000
	PuraMem Flux	-
	PuraMem Performance	450
	PuraMem Selective	410
	NF030105	700
	NF030106	800
	NF030306	380
	NanoPro S-3011	-
	NanoPro S-3012	-
	NanoPro S-3014	-
Acetonitrile	oNF-1	-
	oNF-2	-
	oNF-3	-
	PuraMem Flux	-
	PuraMem Performance	-
	PuraMem Selective	-
	NF030105	-
	NF030106	-
	NF030306	-
	NanoPro S-3011	-
	NanoPro S-3012	-
	NanoPro S-3014	-
Cyrene	oNF-1	850
	oNF-2	800
	oNF-3	-
	PuraMem Flux	-
	PuraMem Performance	540
	PuraMem Selective	540
	NF030105	900
	NF030106	-
	NF030306	-
	NanoPro S-3011	-
	NanoPro S-3012	-
	NanoPro S-3014	-
DMI	oNF-1	850
	oNF-2	800
	oNF-3	-

	PuraMem Flux	-
	PuraMem Performance	620
	PuraMem Selective	600
	NF030105	1000
	NF030106	-
	NF030306	-
	NanoPro S-3011	-
	NanoPro S-3012	-
	NanoPro S-3014	-
GVL	oNF-1	800
	oNF-2	850
	oNF-3	1000
	PuraMem Flux	1150
	PuraMem Performance	550
	PuraMem Selective	500
	NF030105	-
	NF030106	-
	NF030306	-
	NanoPro S-3011	-
	NanoPro S-3012	-
	NanoPro S-3014	-

"-": No MWCO obtained (lower than 90% rejection).

Table S2. Summarise of MWCO (g mol^{-1}) data obtained for the most promising commercial membranes used for green solvent recycling.

Membranes	MWCO Acetone	MWCO EtOAc	MWCO MeCN	MWCO Cyrene	MWCO DMI	MWCO GVL
oNF-1	1070	780	-	850	850	800
oNF-2	950	850	-	800	800	860
PuraMem Performance	550	450	-	540	620	550
PuraMem Selective	750	410	-	540	600	500

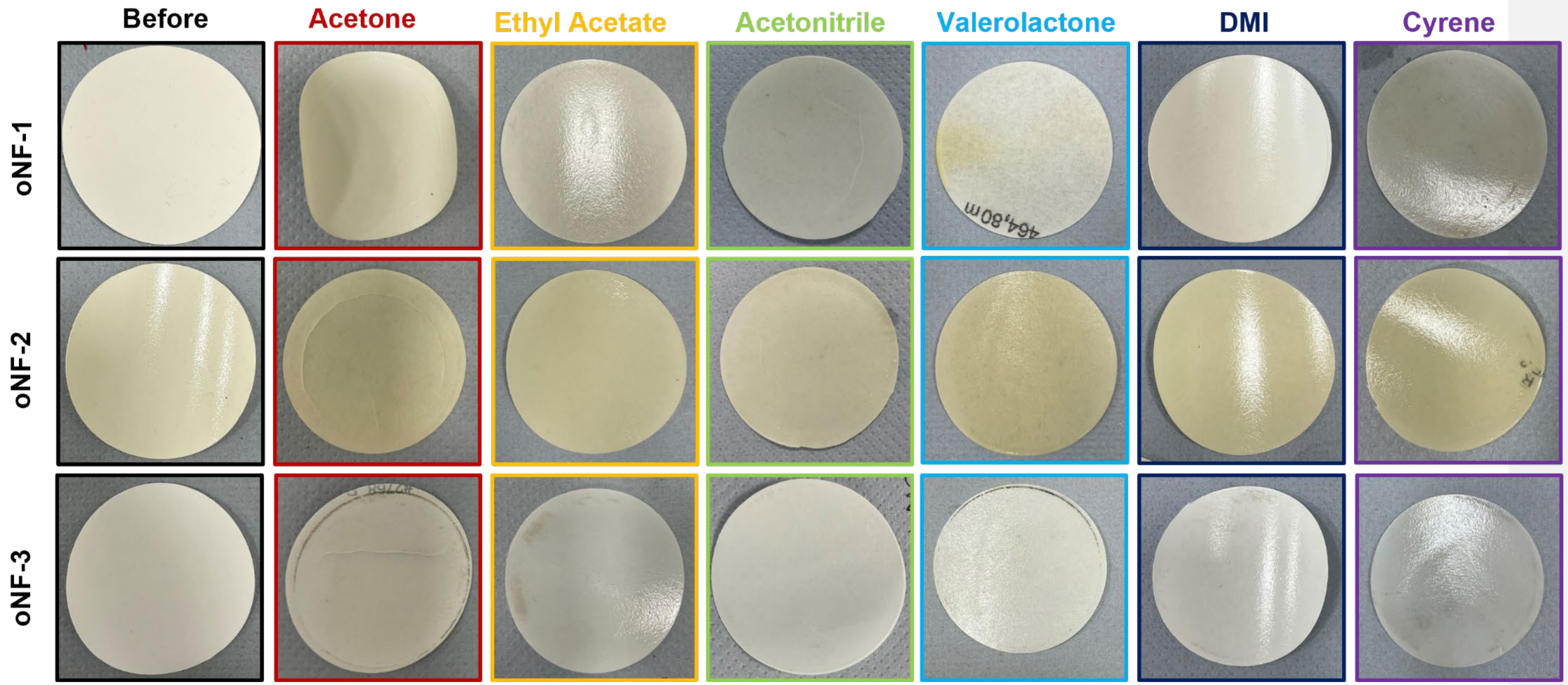

Figure S9. Photos of the Borsig series membranes after being tested in the different solvent for 72h at 30bar.

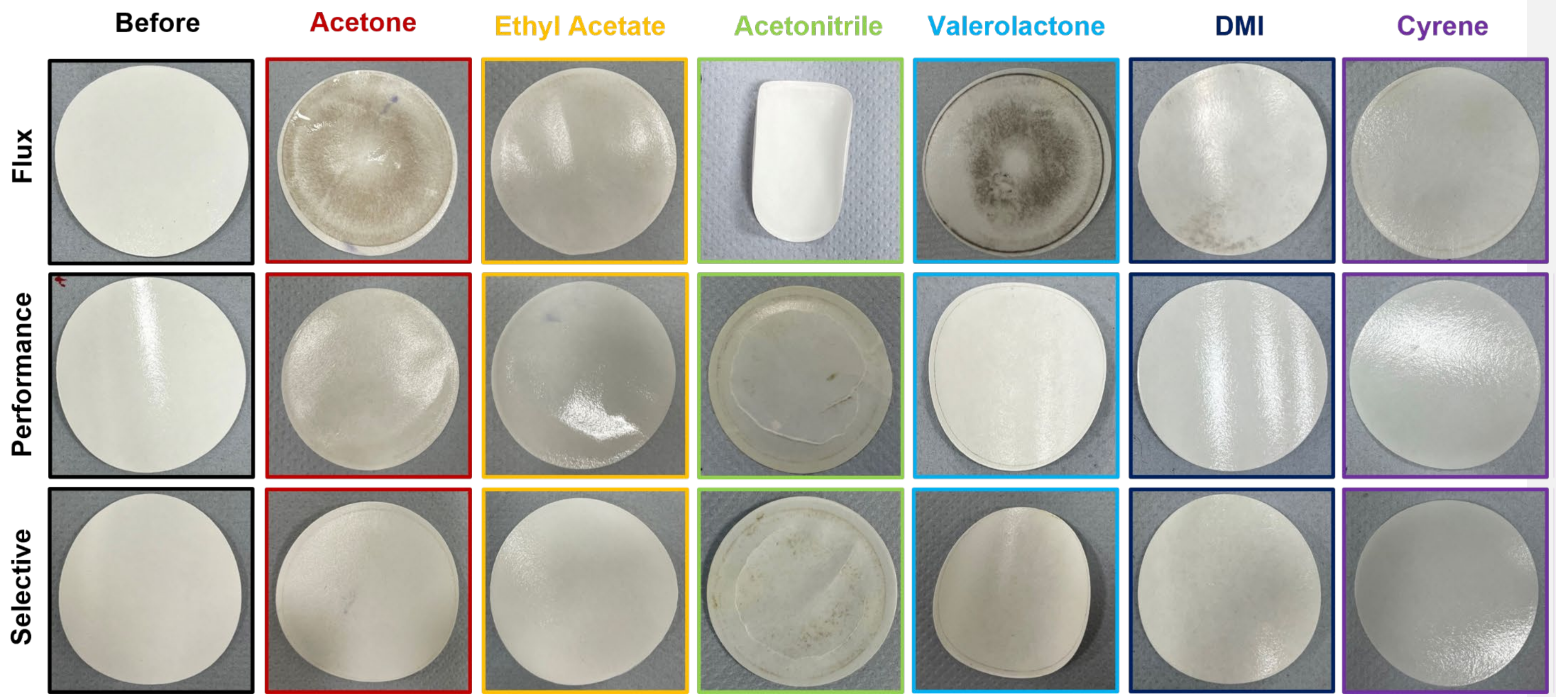

Figure S10. Photos of the Evonik PuraMem series membranes after being tested in the different solvent for 72h at 30bar.

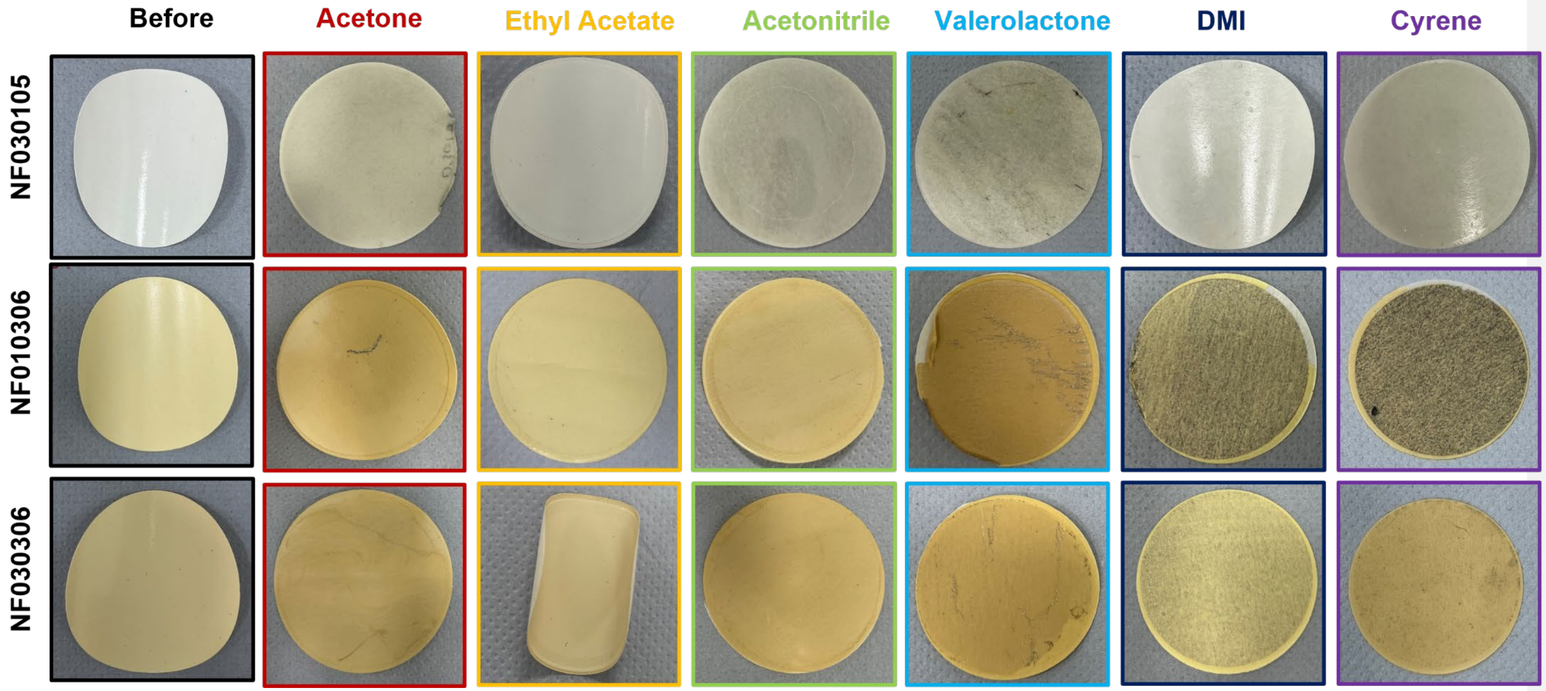

Figure S11. Photos of the Solsep series membranes after being tested in the different solvent for 72h at 30bar.

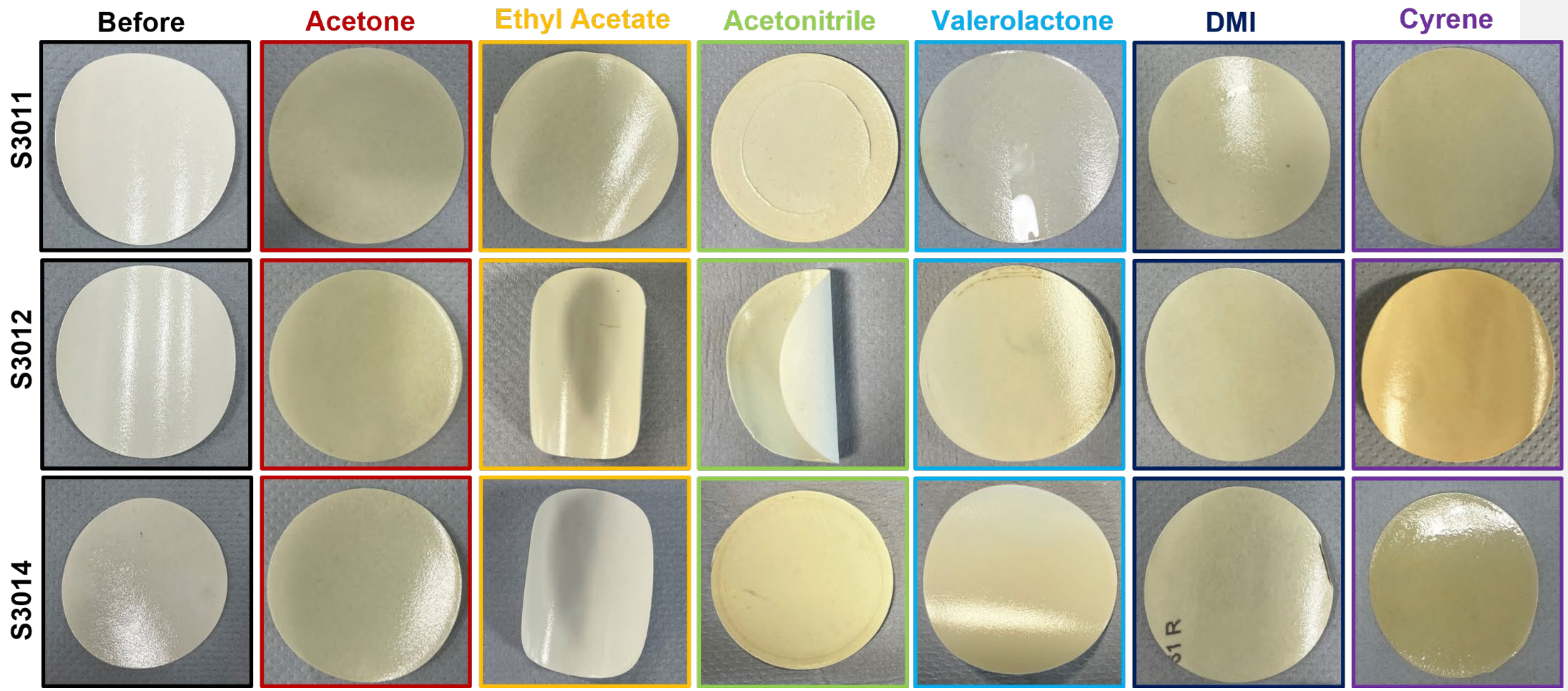

Figure S12. Photos of the AMS series membranes after being tested in the different solvent for 72h at 30bar.

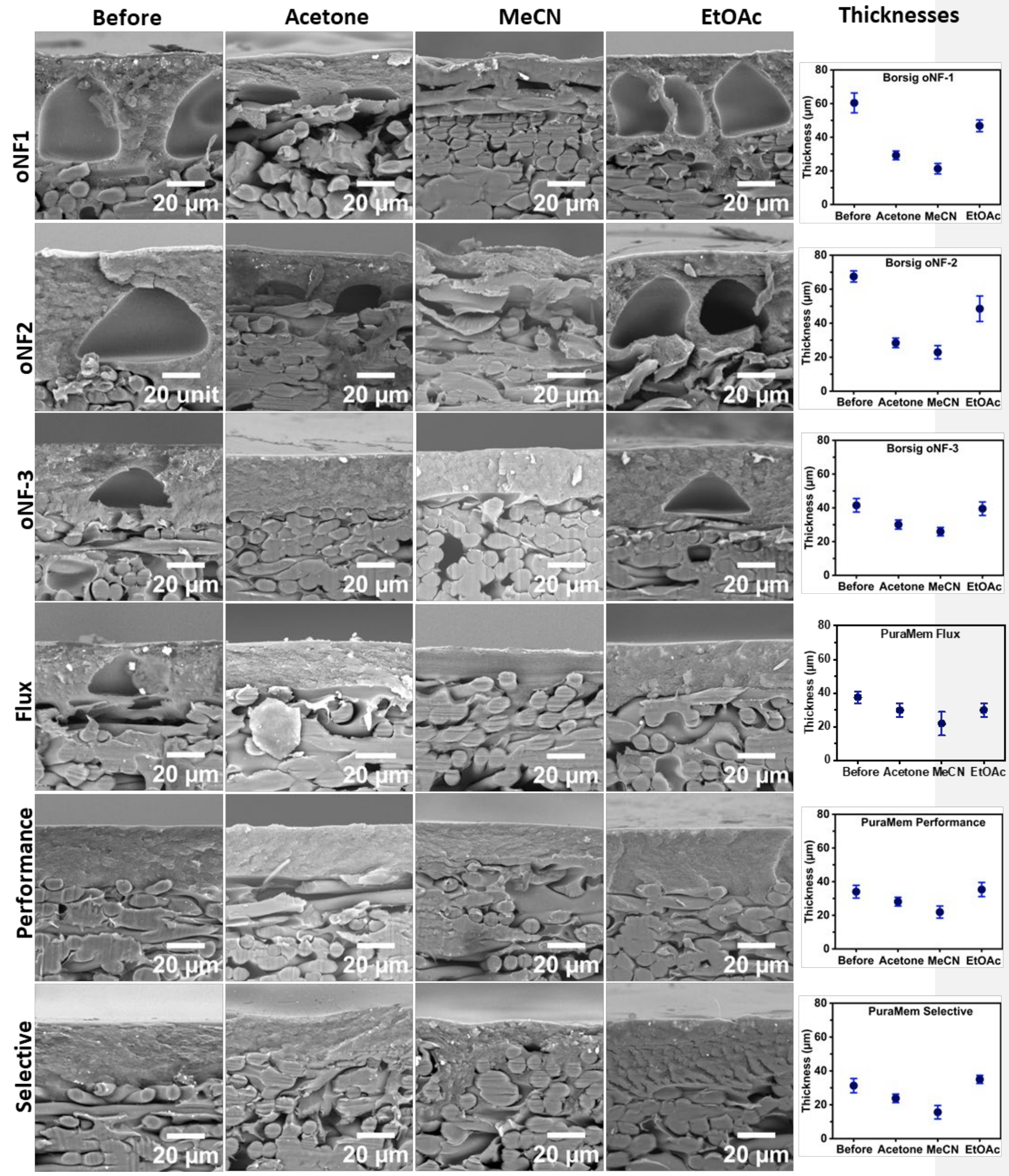

Figure S13. SEM cross section images of Borsig and PuraMem membranes series before and being tested in acetone, acetonitrile and ethyl acetate. With the corresponding graph of their composite top layer thickness depending on the solvent used.

Commented [LP1]: Is this the top layer thickness or total ? I suppose what has been measured is composite PDMS+PAN thickness, she should clarify.

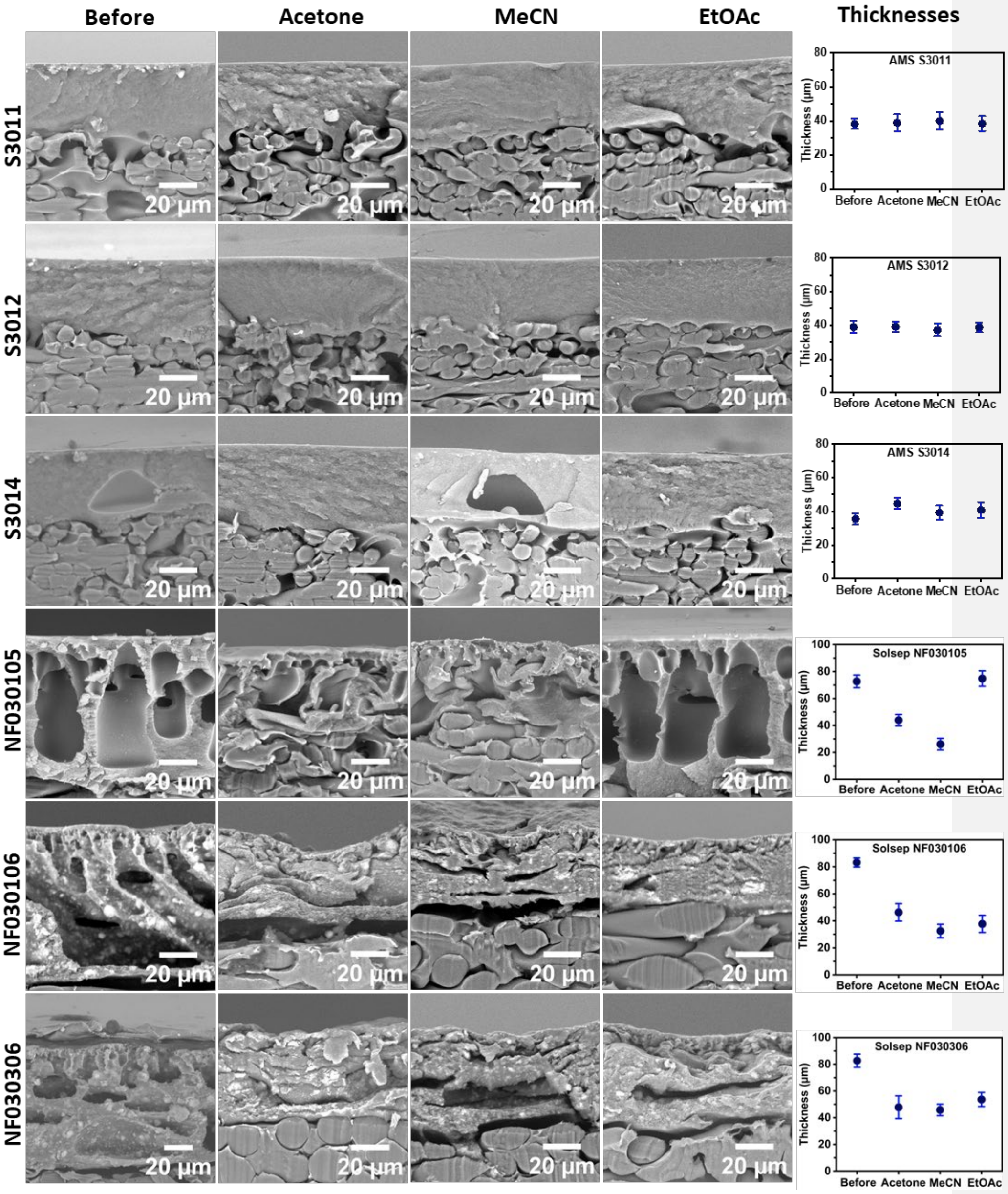

Figure S14. SEM cross section images of AMS and Solsep membranes series before and being tested in acetone, acetonitrile and ethyl acetate. With the corresponding graph of their composite top layer thickness depending on the solvent used.

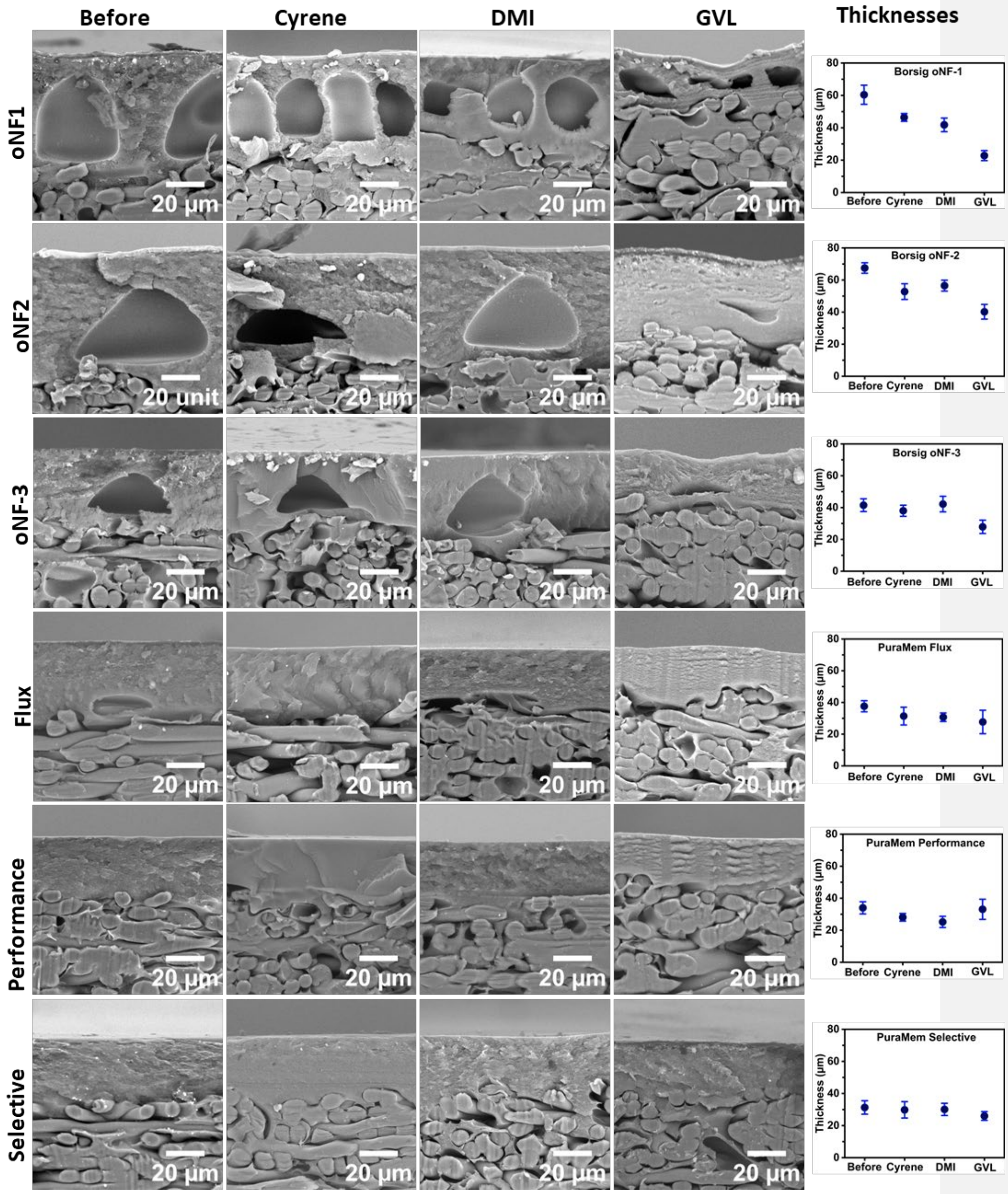

Figure S15. SEM cross section images of Borsig and PuraMem membranes series before and being tested in cyrene, DMI and GVL With the corresponding graph of their composite top layer thickness depending on the solvent used.

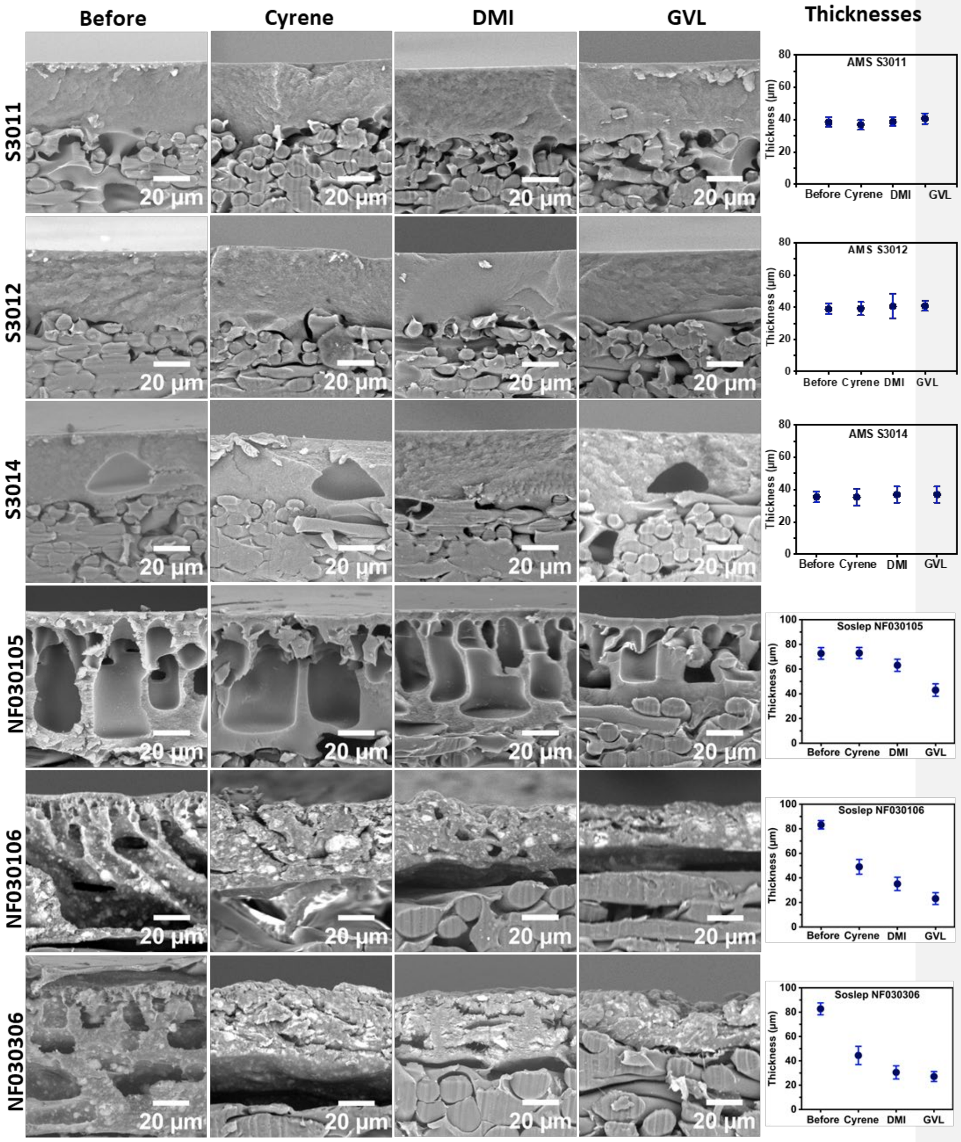

Figure S16. SEM cross section images of AMS and Solsep membranes series before and being tested in cyrene, DMI and GVL With the corresponding graph of their composite top layer thickness depending on the solvent used.

Table S3. Average top layer membranes thicknesses measured by cross-section SEM before and after tested in the green solvents using cross-flow cells.

Membranes \ Solvents Thickness (nm)	Before	Acetone	MeCN	EtOAc	Cyrene™	DMI	GVL
Borsig oNF-1	60.4 ± 5.9	29.2 ± 2.6	21.3 ± 3.1	46.8 ± 3.5	46.4 ± 2.4	41.8 ± 4.2	22.8 ± 3.1
Borsig oNF-2	67.5 ± 3.3	28.4 ± 2.8	22.9 ± 3.9	48.5 ± 7.5	52.8 ± 4.9	56.5 ± 3.4	40.2 ± 4.6
Borsig oNF-3	41.5 ± 4	30.1 ± 2.7	25.9 ± 2.5	39.5 ± 4	38 ± 3.5	42.2 ± 4.9	27.9 ± 4.2
PuraMem Flux	37.6 ± 3.5	29.9 ± 4.2	22 ± 7	31.4 ± 5.6	31.4 ± 5.6	30.7 ± 2.7	27.7 ± 7.4
PuraMem Performance	34 ± 3.8	28.1 ± 2.6	21.9 ± 3.6	35.3 ± 4.2	28.1 ± 2.4	25.2 ± 3.5	33.1 ± 6.3
PuraMem Selective	31.3 ± 4.2	23.8 ± 2.5	15.6 ± 4	35 ± 2.4	29.8 ± 5.1	30.1 ± 3.8	26 ± 2.7
Solsep NF030105	72.7 ± 4.7	43.9 ± 4.2	26.1 ± 4.3	74.8 ± 5.7	73 ± 4.5	63.1 ± 4.9	43 ± 5.1
Solsep NF030106	83.2 ± 3.4	46.3 ± 6.5	32.5 ± 5	37.7 ±6.4	49 ± 6	35.1 ± 5.4	23.1 ± 4.8
Solsep NF030306	82.7 ± 4.9	47.8 ± 8.5	45.8 ± 4.3	53.6 ± 5.3	44.4 ± 7.5	30.5 ± 5.4	27.1 ± 4.1
NanoPro S-3011	38.3 ± 3	39 ± 4.9	40.1 ± 5.3	38.5 ± 4.4	36.8 ± 3	38.7 ± 2.7	40.6 ± 3.3
NanoPro S-3012	39 ± 3.4	39.1 ± 2.8	37.3 ± 3.6	38.7 ± 2.9	39.2 ± 4.2	40.6 ± 7.7	40.9 ± 3.2
NanoPro S-3014	35.5 ± 3.5	44.7 ± 3.3	39.2 ± 4.3	40.8 ± 4.7	35.3 ± 5.1	36.9 ± 5	36.9 ± 5

Table S4. Comparison of the averages permeance values (L m^{-2} $h^{-1}bar^{-1}$) of the membranes in cyrene-Polyols and cyrene-PS mixtures. Average permeances values were calculated after 3 days testing at 30bar.

Membranes	Borsig oNF-1	Borsig oNF-2	PuraMem Performance	PuraMem Selective
Average permeance cyrene-Polyols	0.0046 ± 0.001	0.035 ± 0.008	0.071 ± 0.01	0.054 ± 0.002
Average permeance cyrene-PS	0.047 ± 0.005	0.05 ± 0.01	0.058 ± 0.02	0.038 ± 0.009

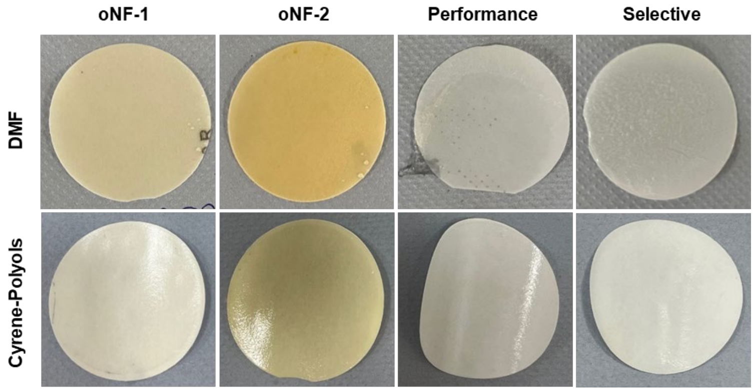

Figure S17. Photos of the oNF-1, oNF-2, PuraMem Performance and PuraMem Selective membranes after being immersed in DMF for 5 minutes (top) and in cyrene-polyols after 72h at 30bar (bottom).

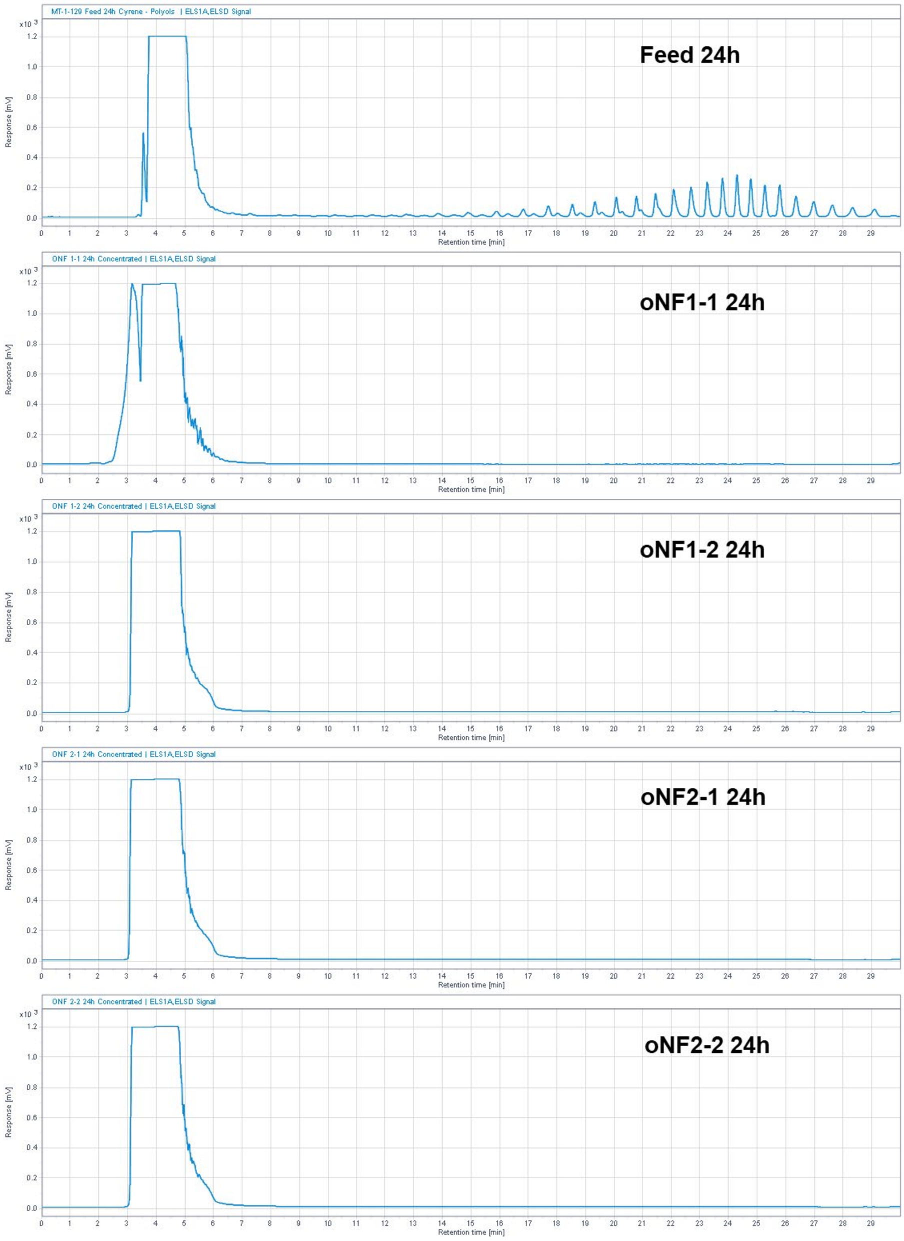

Figure S18. Unprocessed HPLC traces of oNF-1 and oNF-2 membranes tested in cyrene-polyols after 24h.

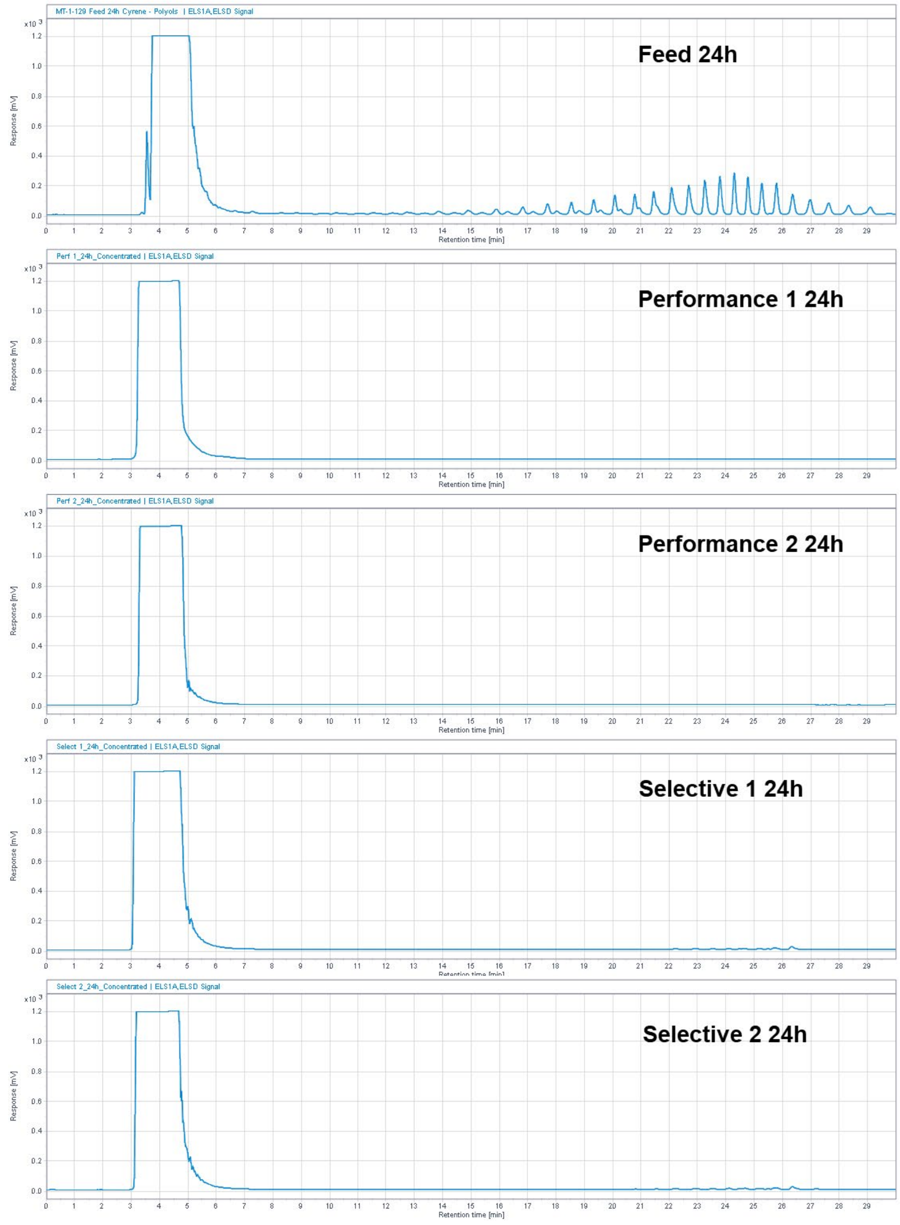

Figure S19. Unprocessed HPLC traces of PuraMem Performance & Selective membranes tested in cyrene-polyols after 24h.

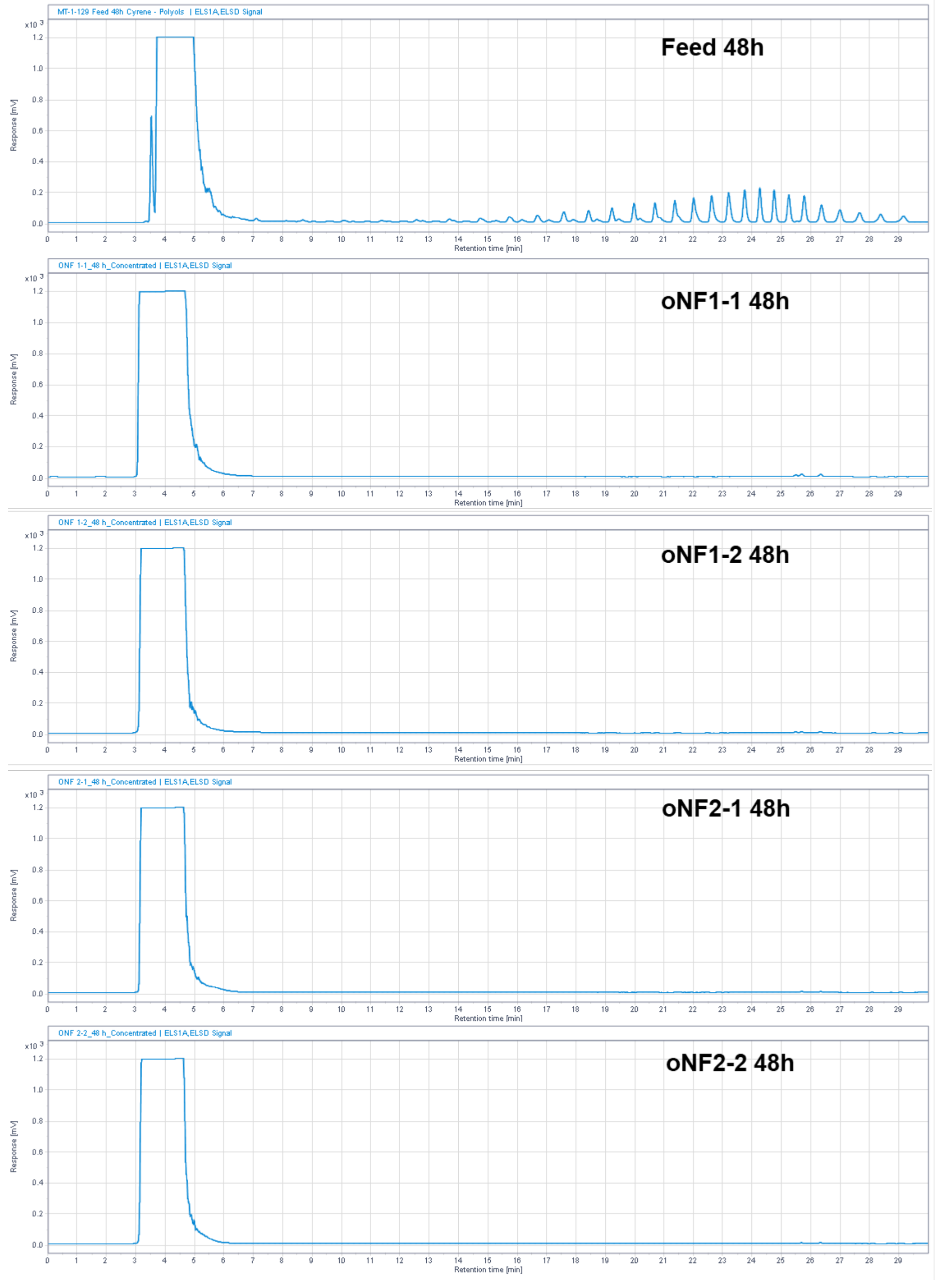

Figure S20. Unprocessed HPLC traces of oNF-1 and oNF-2 membranes tested in cyrene-polyols after 48h.

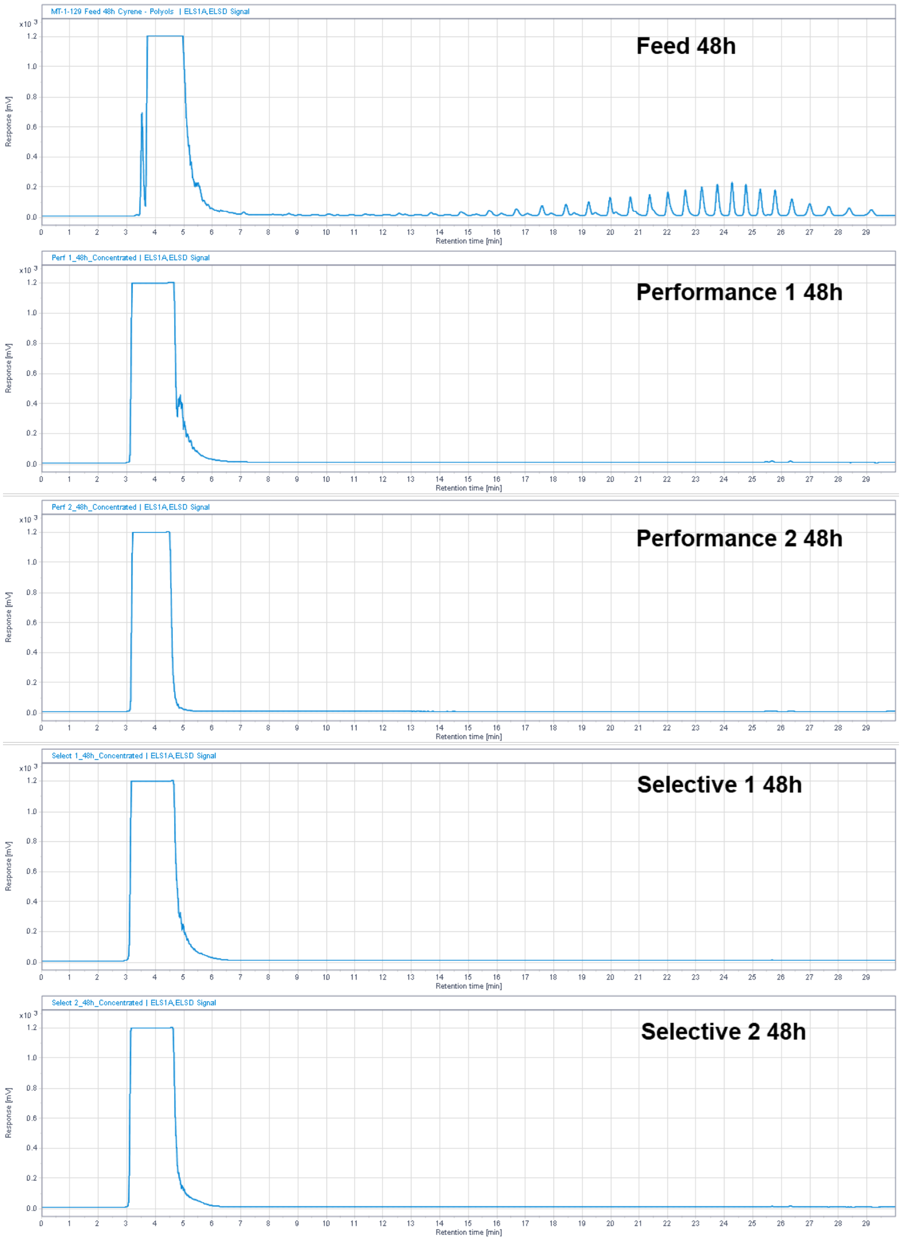

Figure S21. Unprocessed HPLC traces of PuraMem Performance & Selective membranes tested in cyrene-polyols after 48h.

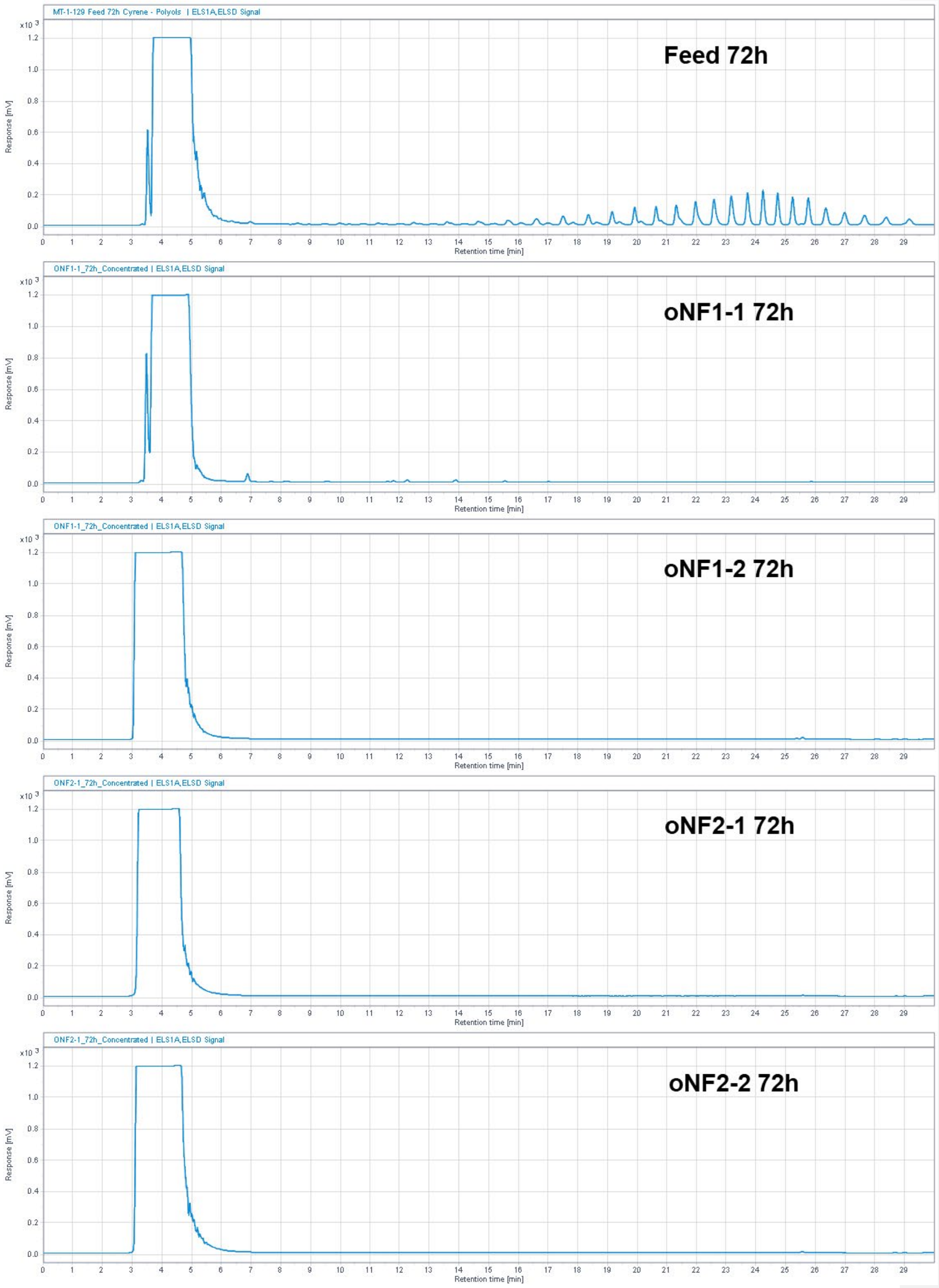

Figure S22. Unprocessed HPLC traces of oNF-1 and oNF-2 membranes tested in cyrene-polyols after 72h.

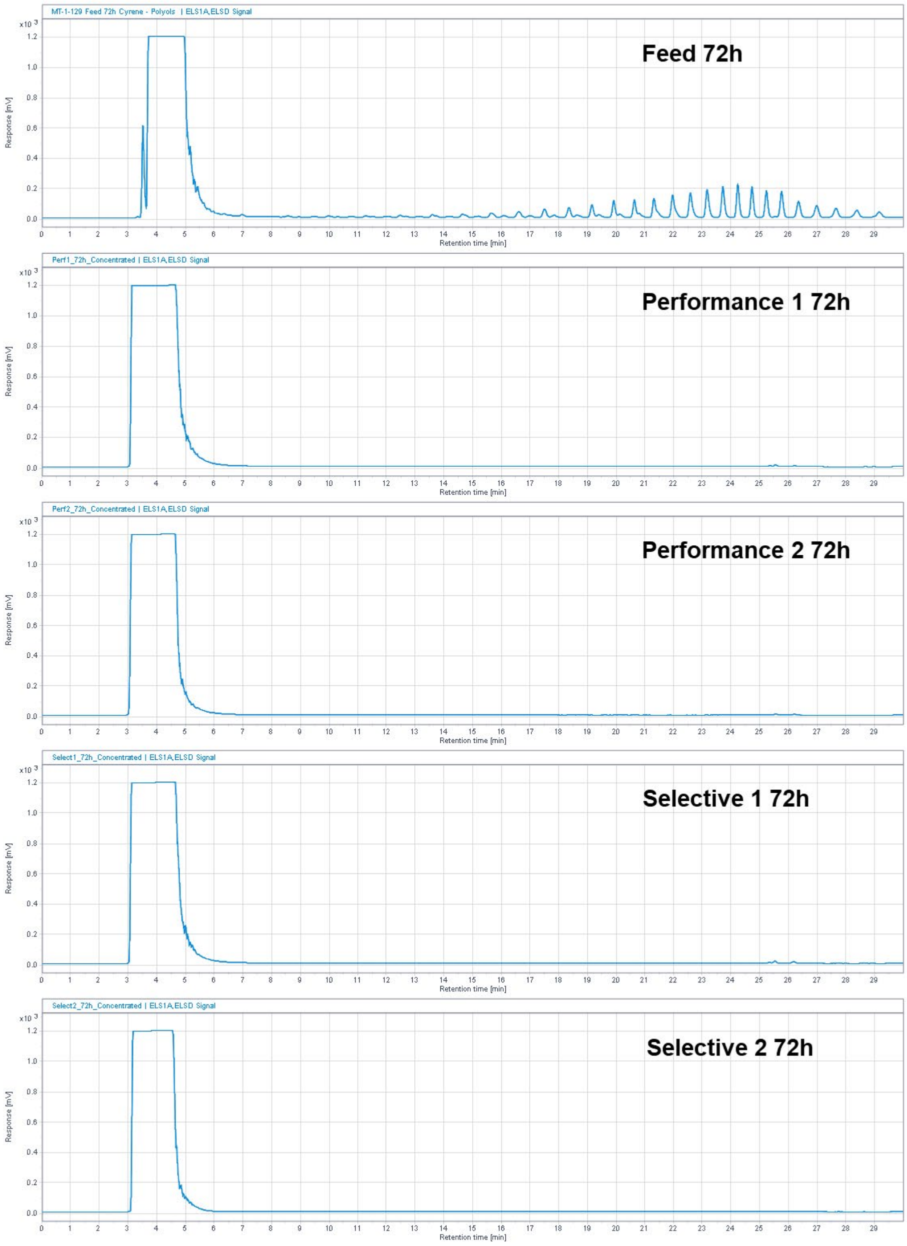

Figure S23. Unprocessed HPLC traces of PuraMem Performance & Selective membranes tested in cyrene-polyols after 72h.
